# Effect of Type of Cow-Calf Contact on Health, Blood Parameters, and Performance of Dairy Cows and Calves

**DOI:** 10.3389/fvets.2022.855086

**Published:** 2022-04-12

**Authors:** Margret L. Wenker, Cynthia M. Verwer, Eddie A. M. Bokkers, Dennis E. te Beest, Gerrit Gort, Daiana de Oliveira, Ad Koets, Rupert M. Bruckmaier, Josef J. Gross, Cornelis G. van Reenen

**Affiliations:** ^1^Animal Production Systems Group, Wageningen University & Research, Wageningen, Netherlands; ^2^Livestock Research, Wageningen University & Research, Wageningen, Netherlands; ^3^Louis Bolk Institute, Bunnik, Netherlands; ^4^Biometris, Wageningen Plant Research, Wageningen University & Research, Wageningen, Netherlands; ^5^Department of Animal Environment and Health, Swedish University of Agricultural Sciences, Uppsala, Sweden; ^6^Department of Bacteriology, Host-Pathogen Interaction and Diagnostics, Wageningen Bioveterinary Research, Lelystad, Netherlands; ^7^Veterinary Physiology, Vetsuisse Faculty, University of Bern, Bern, Switzerland

**Keywords:** suckling, cow-calf separation, maternal care, hematology, biological functioning, calf rearing conditions

## Abstract

Prolonged cow-calf contact (CCC) could potentially improve dairy calf welfare. However, it is currently unknown how different types of CCC affect animals' biological functions. We evaluated health and performance parameters of dairy calves and their dams, where calves: (i) had no contact with their dam (NC), in which the calf was removed from the dam directly after birth (*n* = 10); (ii) were allowed to have partial contact (PC) with their dam, in which the calf was housed in a calf pen adjacent to the cow area allowing physical contact on the initiative of the dam but no suckling (*n* = 18); (iii) were allowed to have full contact (FC) with their dam, including suckling, in which calves were housed together with their dams in a free-stall barn (*n* = 20). Throughout the first 7 weeks postpartum, data were collected on the health status, fecal microbiota, hematological profile, immune and hormonal parameters, and growth rates of calves, and on the health status, metabolic responses, and performance of dams. Overall, FC calves had more health issues (*P* = 0.02) and a tendency for higher antibiotic usage (*P* = *0.0*7) than NC calves. Additionally, FC calves showed elevated levels of erythrocytes, hematocrit, hemoglobin, and leukocytes on day 49 compared to NC calves (*P* < 0.001). Calf fecal microbiota changed over time, and we found preliminary evidence that fecal microbiota is affected by the type of CCC, as reflected by differences in relative abundances of taxa including *Lactobacillus* in FC calves compared to NC and PC calves except on days 7 and 66. The FC calves had a greater average daily gain in body weight than NC and PC calves (*P* = 0.002). Cow health was not affected by the type of CCC, although in the first 7 weeks of lactation FC cows had a lower machine-gained milk yield accompanied by a lower fat percentage than NC and PC cows (*P* < 0.001). These results indicate that full contact posed a challenge for calf health, presumably because the housing conditions of FC calves in this experimental context were suboptimal. Secondly, *ad libitum* suckling leads to higher weight gains and negatively affected milk fat content besides machine-gained yields. More research into strategies to improve cow-calf housing and management in CCC systems is warranted.

## Introduction

Under natural conditions, maternal care has been suggested to be essential for the fitness and survival of cattle offspring (1, 2). However, on most commercial dairy farms, it is standard practice to remove newborn calves from the dam within 24 h postpartum. Then, farmers must care for the calves themselves, hence calf health and survival are chiefly reliant on the quality and quantity of animal care provided by people (3–5). Calf rearing is an important aspect of dairy herd management given that heifer calves are the future replacements for the milking cows. Young stock management practices that enhance calf health and performance can improve the future dairy herd's productivity and longevity (6–8). Nevertheless, dairy farms are sometimes faced with morbidity and mortality rates in young calves that are considered to be high (9–11). This not only has significant economic consequences for farmers (12), but it also raises concern regarding animal welfare (13). Moreover, there is increasing public concern regarding the deprivation of maternal care in young dairy calves, which could pose a threat to the dairy industry's social acceptability (14, 15). Re-introducing prolonged maternal care into the current dairy production system as an alternative rearing practice is receiving increasing interest from various stakeholders (14, 16, 17), and has been proposed to be beneficial for animal health and welfare (18, 19).

Those alternative calf rearing systems that allow dairy cows and their calves to stay in contact for a prolonged period of time, so-called cow-calf contact (CCC) systems, can differ in the type of physical contact between dam and calf and are generally described as full or partial CCC systems (20). Full CCC (i.e., unrestricted physical contact including suckling) typically involves keeping calves together within the herd, which allows cow-calf pairs to express natural behaviors, like suckling and resting in contact. Previous work found that full contact improves calf growth rates (19), positively affects udder health (21), and promotes the expression of natural behavior (22). However, a recent review showed inconsistent and contradictory results for the effect of suckling on calf health, for example, regarding cryptosporidiosis, pneumonia, and mortality. The studies addressing calf diarrhea pointed mostly to beneficial or no effects of suckling (23). From the dairy producers' perspective, there are some major concerns regarding full CCC, such as loss of saleable milk, milk ejection disturbances (e.g., inhibition of milk let-down), and difficulties with calf monitoring (18). Those concerns may be overcome by allowing partial CCC, where contact is restricted by limiting the physical contact and preventing suckling (e.g., housing the calf adjacent to the dams' pen rather than inside the pen) (20). However, to date, no work investigated the consequences of prolonged partial CCC for animal health and performance (i.e., biological functioning). Characterizing important biological systems, such as the gut microbiota (24), and biomarkers, such as cortisol, insulin-like growth factor 1 (IGF-1), cholesterol, and immunoglobulins (25) can provide a deeper insight into the animal's development and disease resistance (26). Overall, there is a need for broader and more systematic investigations before specific recommendations for CCC systems can be made (22).

Given the contradictory literature and existing knowledge gaps regarding the effect of maternal contact on the animals' biological functioning, the objective of this study was to evaluate the effect of different types of CCC on clinical health, blood parameters (i.e., immunological, hormonal, metabolic, hematological profiles), fecal microbiota, and performance of dairy cow and calf.

## Materials and Methods

This study was conducted at the Knowledge Transfer Center in Zegveld (the Netherlands), a dairy research farm, from February 2019 to July 2020. All applicable international, national, and institutional guidelines for the care and ethical use of animals were followed. The experimental design was approved by the Central Committee on Animal Experiments (The Hague, the Netherlands; approval number AVD4010020174307).

### Animals and Experimental Design

In this study, 48 Holstein Friesian cows were included with a parallel-group design. Cows were included at calving when they gave birth to a single heifer calf without substantial calving difficulties or health problems. The mean parity was 2.9, ranging from 1 to 7. In order for calves to have a similarly aged peer, every two cows that calved successively were assigned to the same treatment to have either of the following conditions: (i) no contact (NC) with their calf, in which calves were removed directly after birth and the calf was housed in a calf barn (*n* = 10); (ii) partial contact (PC) with their calf, in which calves were housed in a pen adjacent to the cow area allowing physical contact on the initiative of the dam but no suckling (*n* = 18); (iii) full contact (FC) with their calf including suckling, in which calves were housed together with the dams in a free-stall barn (*n* = 20). The treatment order for every set of two cows was randomized in each block of six successive calvings. Prolonged CCC was allowed for 10 weeks, although from 7 weeks onward, gradual weaning and separation strategies were applied. In the present study, we report data from cow-calf pairs during the first 7 weeks. However, this study was part of a large longitudinal experiment that followed the animals for longer: cows were followed until 12 weeks postpartum, and calves were studied up to 6 months of age. Furthermore, 10 calves per treatment were considered sufficient according to sample size calculations. However, it became necessary to have 10 more cow-calf pairs in two treatments given that two different debonding strategies were investigated in both the PC and FC group^1^ as part of the larger experiment. Due to a twin birth that led to exclusion of two calves, the PC group remained with 18 calves in total.

### Calving Management

Based on signs of imminent calving, cows were moved into an individual indoor straw-bedded maternity pen (3 m wide × 5.1 m long) situated inside the free-stall barn. Cows that were about to calve were continuously video-monitored and the calving was assisted if necessary. Despite regular checks of calving signs by farm staff, eight cows (one NC, two PC, and five FC cows) calved in the dry cow pen but were moved into an individual maternity pen and still included in the trial. Immediately after birth, navels were dipped with 2% iodine and the birth weight of the newborn calf was measured on a full-body calf scale (Type 8700, Welvaarts, the Netherlands). All NC calves were removed from the dam within 1.5 h after birth (median: 6 min, range: 1–63 min) and placed in an individual straw-bedded calf box (Topcalf Duo-Flex, Schrijver, the Netherlands; see Housing and feeding for details) in an indoor calf barn. PC calves were placed in a cuddle-box [consisting of four plywood plates of 1.2 m wide × 0.8 m high; see (27) for details] inside the maternity pen. The cuddle-box prevented suckling, while still allowing tactile, visual, audible, and olfactory contact and was placed in one of the corners across the feeding rack. The cow could lick and sniff her calf when the calf would be standing or lying by moving her head into the box to reach the calf. All FC calves stayed in full contact with their dam inside the maternity pen. When the barn temperature was below 10°C, calves were provided with a heating lamp.

All cows were milked with a mobile milking machine (Mini-milker, Kurtsan, Turkey) twice daily in their pen. In order to reduce the risk of failure of passive transfer, we standardized the first colostrum intake, and all calves were bottle-fed with (mean ± SE) 2.8 ± 0.1 L of colostrum from their own mother within 2 h (± 17 min) after birth regardless the colostrum quality. Calves in the NC and PC treatment group received an additional 2 L colostrum by the bottle at 8 to 12 h as well as at 20 to 24 h after birth. After the first colostrum meal by the bottle, FC calves could suckle the remaining colostrum directly from the dam's udder. To check colostrum quality, the Brix value of the first colostrum meal was measured using an optical refractometer (0–32%, Bio Enterprise B.V., the Netherlands). In brief, seven FC calves (but none of the NC or PC calves) started to suckle their first colostrum before farm staff could bottle-feed them. However, four of them did still drink colostrum from the offered bottle as well. All NC cows returned to the designated group pen in the free-stall barn after the second postpartum milking. Both PC and FC calves stayed with their dam in the maternity pen for about 72 h, after which they moved to their designated group pens in the free-stall barn.

### Housing and Feeding

**Figure 1:**
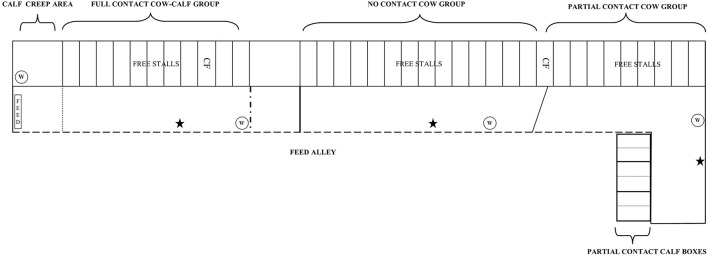
Experimental cows were housed in three dynamic group pens inside a free-stall barn [i.e., full contact (FC), no contact (NC), and partial contact PC group pen]. Within the FC group pen, FC calves were kept with the dams in the group pen but had access to a calf creep area with a straw-bedded lying area, water bucket, hay and concentrates. A metal bar hindered cows to access this area. PC calves were housed individually in a straw-bedded calf box for the first 2 weeks, after which they were pair housed in the same box with their similar-aged peer. In each individual calf box ad lib water, hay, and concentrates were provided. The calf boxes were placed behind a wall (1.2 m high) to limit physical contact and prevent suckling. NC calves were housed in an indoor naturally ventilated calf barn in similar pens as PC calves and were also pair housed at 2 weeks of age. W, water; CF, individual concentrate feeder, ⋆ = automated cow brush.

The experimental cows were kept in dynamic groups separate from the rest of the farm herd in an indoor free stall barn in three different group pens (i.e., NC, PC, and FC group) (Figure 1). The free-stall barn was naturally ventilated with open sidewalls and had perlite-bedded lying stalls (1.1 × 3 m). The closed floor was covered with rubber and cleaned 8 times a day by an automated scraper. All experimental cows were milked twice a day at ~8:00 am and 6:00 pm in the milking parlor with a five-point open tandem side and 11 side-by-side places. Cows were fed grass silage (early spring cuttings) once a day at ~9:30 am Feed was pushed automatically (MoovPro, JOZ, the Netherlands) to the feeding rack 8 times a day. Additionally, cows could eat up to 10 kg of concentrates per day that were provided partly in the milking parlor and by an individual concentrate feeder. When the barn temperature was below 10°C, all young calves were fitted with a calf jacket for the first 3 weeks of life.

All NC calves were kept in a straw-bedded calf box (Topcalf Duo-Flex, Schrijver, the Netherlands) in an indoor naturally ventilated calf barn separate from the free-stall barn. One calf box could house two calves individually (1 × 1.6 m), but also offered the opportunity to pair house them (2 × 1.6 m) by removing the partition wall in the middle of the box. The NC calves were housed individually for the first 2 weeks, after which they were pair housed with their similar-aged peer. Each calf was provided with ad-lib water, hay, and concentrates (Topfok Kalf, de Samenwerking, the Netherlands) from 3 days of age onwards.

All PC calves were kept in similar calf boxes as NC calves, but were housed inside the free-stall barn behind a wall (1.2 m high) adjacent to the PC cow group pen. This set-up prevented suckling, direct contact with manure of adult cows, and housing calves within the cow herd, while it allowed for individual feeding of calves, as well as visual, auditory, olfactory, and limited tactile contact between cow-calf pairs [see (27) for an illustration]. Cows could move their head over the wall and when the calf was standing, cow-calf pairs could sniff and lick each other. The PC calves were also housed individually for the first 2 weeks, after which they were pair housed in the same box with their similar-aged peer. Adlib water, hay, and concentrates were provided from 3 days of age onwards to each individual calf. The PC group never exceeded more than six cow-calf pairs.

All FC calves were housed together with the dams in the FC group pen in the free-stall barn but had access to a calf creep area (inaccessible for the dams). The calf creeps area (3.3 × 4.8 m) provided them with a straw-bedded lying area (3.3 × 1.9 m) and ad-lib water, hay, and concentrates from the day the newborn calves moved into the free-stall barn. The FC group never exceeded more than eight cow-calf pairs.

For NC and PC calves, bulk tank milk was provided in individual teat buckets following a fixed feeding schedule (Table 1), afterward, three colostrum meals were consumed. Milk was provided around 8:00 am, 1:00 pm, and 6:00 pm. Bulk tank milk was heated up to 41°C using a milk taxi (Milchtaxi 2.0, Holm & Laue, Germany) before being fed to the calves. The amount of daily milk intake of NC and PC calves was recorded after every fed meal. The FC calves could suckle their dams and, if allowed, other dams throughout the whole day excluding milking hours.

**Table 1:** Fixed feeding schedule for each individual calf with no contact or partial contact to their dam-fed bulk tank milk.

**Week of age**	**Number of meals per day**	**Amount of milk per meal (L)**
1	3	2.5
2	3	3.0
3	3	3.5
4	3	3.5
5	2	3.5
6	2	3.0
7	2	2.0
8	1	1.0

### Data Collection

#### Clinical Health Assessment

Once a week all calves between the age of 4 and 49 days were clinically assessed by trained observers using a standardized health scoring system (Table 2). The health scoring system was adapted from recent work on clinical health indicators for calves (28) to evaluate the respiratory system (nasal discharge, ocular discharge, cough), fecal consistency, navel inflammation, and rectal temperature on a 4-point scale. Use of antibiotics and other medicine plus any observed health problems were recorded by the farm staff in a daily logbook for both cows and calves during the entire experiment.

**Table 2:** Description of health parameters scored on a weekly basis during the first 7 weeks of life [adapted from (28)].

**Variable**	**Score**			
	**0**	**1**	**2**	**3**
Nasal discharge	Normal serous discharge	Small amount of unilateral discharge	Moderate amount of bilateral discharge	Copious, bilateral mucopurulent discharge
Ocular discharge	Normal	Small amount of ocular discharge	Moderate amount of bilateral discharge	Heavy ocular discharge
Cough^a^	No cough	Induced single cough	Induced repeated coughs or occasional spontaneous cough	Repeated spontaneous cough
Fecal consistency	Normal consistence	Pasty, semi-formed	Pasty with large amounts of water, content adhered in the perineum and tail	Liquid with fecal content adhered in the perineum and tail
Navel inflammation	Normal	Slightly enlarged, not warm or painful	Slightly enlarged with slight pain or moisture	Enlarged with heat, pain or malodorous discharge
Rectal temperature (°C)	37.8–38.2	38.3–38.8	38.9–39.4	>39.4

a *Cough was induced by trachea palpation*.

#### Performance Measures

During the weekly health assessment, calf body weight was measured using a full-body calf weighing scale (Type W8700, Welvaarts, the Netherlands). Additionally, heart girth and back length were measured with a tapeline and hip height was measured using a rod (Kerbl, Germany). At 6 months of age, the bodyweight of all calves was recorded once more using a full-body cow scale (Type 8700, Welvaarts, the Netherlands).

For the assessment of cow performance, data on machine-harvested milk yield and the moment of the first insemination of experimental cows were automatically collected using AgroVision dairy farm management software (AgroVision B.V., the Netherlands). Moreover, milk composition was evaluated based on milk samples collected every 3 weeks year-round. In addition to the percentage of milk fat, protein, and lactose, the somatic cell count (SCC) was analyzed (ISO 9622 and ISO 13366-2, Qlip, the Netherlands).

#### Blood Sample Collection and Analysis

##### Passive Transfer of Immunity

Blood samples (9 ml) from calves were taken via jugular venipuncture at 24–48 h of age into citrate vacutainer tubes (Vacuette, Greiner BioOne, Austria). Samples were centrifuged for 20 min at 2,000 rpm and 4°C right after collection, and plasma was stored at −20°C until further processing. Immunoglobulin (Ig)G concentrations were measured in plasma samples with an indirect bovine IgG-specific ELISA. Wells were coated for 1 h with affinity-purified sheep anti-bovine IgG-heavy chain (Cat. No. A10-118A-13, Bethyl Laboratories, USA) diluted at 1:100 in coating buffer (0.05 M carbonate-bicarbonate, pH 9.6, Merck KGaA, Germany). Plates were washed 6 times with 50 mM TRIS.14 M NaCl (Merck KGaA, Germany), incubated for 1 h in the same buffer (blocking), and then washed 6 more times. After the 6th wash, 24 mg/ml of bovine reference serum (Cat. No. RS10-103-5, Bethyl Laboratories, USA) or diluted calf sera were added to each well, and the plates were incubated for 1 h. Wells were then washed 6 times, 100 ml of sheep anti-bovine IgG-heavy chain (1:120,000) conjugated to horse-radish peroxidase (HRP) (Cat. No. A10-188P-30, Bethyl Laboratories, USA) were added, and plates were incubated for 1 h. After incubation, plates were washed 6 times and tetramethyl benzene (TMB) (SanBio B.V., the Netherlands) was added. Reactions were stopped after 15 min with 0.2 M H2SO4 (Merck KGaA, Germany), and the optical density at 450 nm was determined with an automated plate reader. The standard curve was generated by means of a 4-parameter curve fit and the IgG concentrations in the test samples were quantified by interpolating their absorbance from the standard curve generated in parallel with the samples.

##### Hematology

For the assessment of calves' hematological profile, blood samples (9 ml) was taken *via* jugular venipuncture 24–48 h, 14 days, and 49 days of age into EDTA vacutainer tubes (Vacuette, Greiner BioOne, Austria). Calf age at the actual sample moment could deviate from the intended 14 and 49 days of age (ranging from −6 to +6 days for both time points), as the majority of calves were sampled during the weekly health and growth assessments. We followed this approach to reduce the handling of calves, as the animals' response to humans was also studied in another part of this experiment. Samples were stored and transported at 4°C prior to the analyses. Fluorescence flow cytometry (European Veterinary Laboratory, the Netherlands) was used to determine absolute numbers of different cell types in full blood, including cell count for leukocytes (WBC), granulocytes (GRA), lymphocytes (LYM), less frequently occurring, and rare white blood cells (MID), erythrocytes (RBC), platelets (PLT), procalcitonin (PCT), percentage of basophils, neutrophils, eosinophils, lymphocytes, monocytes, and erythrocyte indices like hematocrit (HCT), mean corpuscular volume (MCV), hemoglobin (HGB), mean corpuscular hemoglobin (MCH) and mean corpuscular hemoglobin concentration (MCHC), mean platelet volume (MPV), and red cell distribution width (RDW).

##### Immunoglobulins and Hormones

To assess calves' natural autoantibodies (N-IgA, N-IgG, and N-IgM titers), and concentrations of cortisol, IGF-1, cholesterol, and insulin at 14 and 49 days of age, blood was collected in different vacutainer tubes. The EDTA samples were stored at 4°C for a maximum of 2 h, whereas serum samples were stored at room temperature for 1 h prior to processing. All samples were centrifuged for 15 min at 3,000 rpm and 4°C and were stored at −20°C until analysis.

Titers of N-IgG, N-IgM, and N-IgA were measured in serum samples with indirect ELISA against phosphorylcholine conjugated to bovine serum albumin (PC-BSA) according to previously published methods (29, 30). Pre-diluted samples (1:10) in PBS mix (PBS + 1% horse serum (HS) +0.05% tween) were coated with different amount of PC-BSA (PC-1011-10, Bioresearch Technologies, Canada): 1 μg/ml for N-IgG and 0.25 μg/ml for N-IgM and N-IgA. N-IgG and N-IgM were detected using 1:20000 diluted sheep polyclonal anti-bovine IgG-heavy chain conjugated to horseradish PO (Cat. No. A10-100P, Bethyl Laboratories, USA), and 1:20000 diluted rabbit polyclonal anti-bovine IgM conjugated to horseradish PO (Cat. No. A10-100P, Bethyl Laboratories, USA). N-IgA was detected using 1:10000 diluted sheep polyclonal anti-bovine IgA conjugated to horseradish PO (Cat No. A10-131P, Bethyl Laboratories, USA). Starting dilution of standards was 1:160 for N-IgG, 1:80 for N-IgM, and 1:20 for N-IgA. Serial dilutions for N-IgG, N-IgM, and N-IgA in serum samples started at 1: 40 (4 steps). After the last 1.5 h incubation at room temperature with the conjugates, plates were washed with demi-water. Each well of the plate was filled with 100 μL of substrate TMB (Sigma Aldrich Chemie, Germany), which contained Milli-Q water, 1% TMB, and 10% TMB buffer. Plates were then incubated for 30 min at room temperature. After the incubation, the reaction was stopped by adding 50 μL of 2.5 N H2SO4 solution to each well. Extinctions were measured with a Multiskan reader (Lab Systems, Finland) with a wavelength of 450 nm. Titers were calculated based on log2 values of the dilution that gave extinction closest to 50% of Emax (31), where Emax represents the highest mean extinction of standard positive serum present on each plate.

Hormones in blood plasma were measured by a radioimmunoassay (RIA) adapted from (32, 33). Plasma insulin concentrations were measured with a homologous double-antibody system using 25.7 IU/mg bovine insulin (Sigma, USA) for standards and for iodination and guinea-pig anti-bovine insulin (#5506, lot GP23; Bioyeda, Weizmann Institute, Israel). Precipitating anti-guinea-pig γ-globulin (Calbiochem, USA) was used as a second antibody to separate antibody-bound from the free hormone. Plasma samples were diluted (1:10) with assay buffer and ovalbumin (35 mg/ml) and paralleled with the standard curve. The intra-assay CV was 9.4% and the inter-assay CV was 5.3%.

Plasma IGF-1 concentrations were measured by extracting 50 μL plasma with 250 μL absolute ethanol and 12.5 μL of 2.4 M formic acid. Recombinant bovine IGF-1 was used as standard and for iodination. A monoclonal antibody against human IGF-1 raised in mice hybridoma cells was used as the first antibody together with normal mouse serum. Sheep-anti-mouse serum (100 μL) was used together with 1,000 μL 6% polyethyleneglycol to separate antibody-bound and free hormone. Plasma from a calf was diluted, so IGF-1 concentrations paralleled the standard curve. The intra-assay CV was 6.4% and the inter-assay CV was 4.3%.

Plasma cortisol concentrations were analyzed by extracting 0.1 ml plasma with 1 ml absolute ethanol. After mixing the tubes on a vortex mixer, the protein precipitate was sedimented by centrifugation at 1,500 × g for 20 min at 4°C. Supernatants were decanted into fresh tubes, evaporated to dryness, and reconstituted in 0.5 ml PBS (0.14 M sodium chloride and 0.01 M sodium phosphate, pH 7) containing 0.1% gelatin. A standard curve was run in duplicate by adding cortisol at concentrations between 0.25 and 100 ng/ml. Upon addition of 0.1 ml diluted antiserum and 0.1 ml [1, 2–3H] cortisol (78 Ci/mmol), each tube was mixed and incubated at 4°C for 15 h. Separation of the free hormone from the bound hormone was achieved by adding 0.4 ml of a 0.75% dextran-coated charcoal suspension. After 4 min, tubes were centrifuged (2,800 × g, 15 min, 4°C) and 0.7 ml were pipetted from the supernatant and mixed with scintillation fluid for radioactivity counting. The intra-assay CV was 9.7% and the inter-assay CV was 6.3%.

Total cholesterol concentrations in blood serum were measured with a commercially available enzymatic kit (Cat. No. Cholesterol FS 1.1350 99 10 021; DiaSys Diagnostic Systems GmbH, Germany) with an autoanalyzer (Cobas Mira, Switzerland).

##### Metabolic Status

Blood samples of cows were taken from the coccygeal vein by a veterinarian at 2–21 days before the expected calving date and at 30–50 days after calving. Sera were tested by the GD Animal Health Service (Royal GD, the Netherlands) for haptoglobin, non-esterified fatty acids (NEFA), beta-hydroxybutyrate (BHBA), magnesium, and urea levels to assess the metabolic status of cows in the prepartum stage (Test package for the dry period, number: 11682) and for calcium, BHBA, and urea levels in the postpartum stage (Test package for the fresh period, number: 11508).

#### Microbiota Sampling and Analysis

Rectal feces samples of calves were collected at 7 days, 28 days, 49 days, and 66 days of age. As mentioned earlier, calf age at the actual sample moment could deviate from the intended age (ranging from −4 to +3 days for the first three sample moments and from −9 to +9 days for the last sample moment), as calves were sampled during the weekly health and growth assessments. Calves were rectally finger-stimulated with sterile-gloved hand to facilitate the collection of at least 5 g feces into a 50 ml polypropylene conical bottom test tube (Cellstar, Greiner BioOne, Austria). Samples were stored at −20°C until analysis.

Total DNA was extracted from 0.2 g fecal samples using QIAamp Fast DNA Stool Mini Kit (QIAGEN, Art. No. 51604, Germany) according to the manufacturer's instruction. In addition, after resuspending the samples in InhibitEx, buffer samples were subjected to repetitive bead-beating (3 times for 30 s with 5 s cooldown in between) using Lysing Matrix B tubes (MP Biomedicals, Art. No. 116911050-CF, USA) and the FastPrep-24 instrument (MP Biomedicals, USA). Microbial DNA extracts were checked on a 2200 Tapestation (Agilent Technologies, USA).

Bacterial community composition was assessed by sequencing the combined V3–V4 hypervariable region of the 16S rRNA gene as previously published (34). Briefly, this region was first amplified by 25 cycles of PCR using the primers CVI_V3-forw CTACGGGAGGCAGCAG and CVI_V4-rev GGACTACHVGGGTWTCT. PCR products were checked on a 2200 Tapestation, and sequencing was performed using a V3 paired-end 300 bp sequencing on a MiSeq sequencer (Illumina Inc., USA). Negative controls were used in each round of amplification to confirm the sterility of reagents, and a mock community bacterial community was included in the sequencing run as a control. More details on sequence processing and bioinformatic analysis can be found in Supplementary Material 1.

#### Hair Samples and Analysis

Hair samples of calves were collected on the day of birth, day 21, and day 56 of age. All samples were collected from the tip of the tail by carefully clipping 2 to 3 cm of the tail hair with surgical scissors as close to the skin as possible (35). The hair samples were stored in individually identified zip-lock plastic bags, which were kept at −20 degrees until further processing. Samples were mechanically cleaned and defatted with 5 ml of n-hexane/isopropanol. Samples were dried overnight at room temperature. The dried samples were cut into small fragments ~1–2 mm with scissors. Individual 100 mg aliquots from each of the samples were milled at 30 Hz with 3 mm beads for 5 min using a TissueLyserII (Qiagen, Germany). The milled hair samples were placed in a glass test tube along with 5 mL of methanol, and the tubes were incubated at 50°C for 18 h. After centrifuging, the liquid in the tubes was transferred to another glass vial and evaporated to dryness in a stream of nitrogen. The remaining residue was dissolved in 200 μL of Neogen extraction buffer. Extraction of all hair samples (0.5 g each) was performed with 100% methanol, after which hair cortisol metabolites were determined using a Neogen cortisol kit (Product nr. 402710, Neogen, USA).

### Data Handling

#### Total Health Score

A total health score (THS) was calculated based on the clinical health assessment (calf age 1–7 weeks), summarizing disease length and intensity for each calf [adapted from (36)]. On a weekly basis, calves were classified for having clinical symptoms of common calf diseases, i.e., respiratory issues [“yes” when they had a composite respiratory score ≥ 4 (based on the sum of ocular discharge, nasal discharge, cough score)], neonatal diarrhea [“yes” when fecal score ≥2 (this category was included for the first 4 weeks of life as an indication of neonatal diarrhea, and comprised either infectious diarrhea or feeding-related loose/liquid manure)], navel inflammation [“yes” when navel score ≥ 2], and fever [“yes” when they had a rectal temperature score of 3]. Subsequently, all clinically detected health problem scores between weeks 1–7 were added to one total score (i.e., THS, dimensionless) per calf. Calves with a low THS (with few clinical symptoms) were in good health, whereas calves with a high THS suffered from more health problems or had a slower recovery throughout the first 7 weeks of life.

#### Growth Rates

Average daily gain (ADG) of calves was calculated for the body weight, as well as increases in hip height, back length, and heart girth between the age of 1–7 weeks.

#### Milk Production

Since milk samples were collected triweekly and cows left the experiment at 12 weeks postpartum, milk yield and composition plus SCC data were averaged per cow during the first 7 weeks of lactation.

### Statistical Analysis

All statistical analyses were performed using SAS (version 9.4, SAS Institute, Institute Inc., Cary, NC, USA), except for the microbiota analysis that was performed using R [version 4.05 (37), Austria]. The animal was treated as the experimental unit. All variables and model residuals were visually checked for normality and homogeneity of variance, and response variables were log-transformed when needed.

#### Growth Rates

Increases in hip height, back length, and heart girth, besides ADG for body weight, were analyzed with a linear mixed model (using SAS procedure PROC GLIMMIX) for continuous data. The systematic part of the model (referred to as model 1) consisted of the following fixed effects:


(1) $$\begin{array}{l} \mu +{\text{Treatment}}_{\text{i}}+{\text{Batch}}_{\text{j}}+{\text{Parity}}_{\text{k}}+\left({\text{Treatment}}_{\text{i}}\times {\text{Parity}}_{\text{k}}\right) \end{array}$$

Here, μ was a base level and Treatment_i_ = type of CCC (i = no contact, partial contact, full contact), Batch_j_ = 16-week time period in which a calf was born (j = 1, 2, 3, 4), and Parity_k_ = parity of the dam (k = primiparous or multiparous) were main effects. Batches were defined retrospectively to control for seasonal differences and varying group sizes in the treatment groups over time. Hence, the duration of the experiment was split up into batches of 16 weeks based on calving dates, so that every treatment was represented in a batch and batches represented the various seasons. Although in each treatment group both primiparous and multiparous animals occurred, parity was unevenly distributed. Given that parity is known to affect calf's growth, health, and colostrum characteristics (38–40), parity and the two-way interaction between parity and treatment were included in the statistical model. Interactions that were not significant (*P* ≥ 0.05) were excluded from the model. In addition, the model comprised a random effect for the interaction between treatment and batch. For all fixed effects, approximate F-tests were used (41) and significance was declared at *P* < 0.05. Subsequent pairwise comparisons were made according to the Tukey method.

#### Total Health Score and Antibiotic Use

Data on the THS of calves were analyzed with the same linear mixed model as for growth rates (see model 1). Due to low prevalences (<5%) in some treatment groups for the percentage of calves classified with clinical symptoms of specific health variables and the percentage of calves treated with antibiotics, those parameters were analyzed using Fisher's exact method for pairwise comparisons.

#### Immunoglobulins

To assess passive transfer of immunoglobulins after colostrum feeding, serum bovine IgG concentrations and colostral brix scores were analyzed with a linear mixed model identical to model 1. For one FC calf, there was a missing IgG value, as the calf was not sampled 24–48 h after birth. Failure of passive transfer was analyzed using Fisher's exact method for pairwise comparisons considering the few incidences.

For continuous data on natural autoantibodies (i.e., N-IgM, N-IgA, N-IgG) in serum samples of calves at 14 and 49 days of age, a total of 8 out of 96 samples were missing. Those serum samples could not be collected due to issues with blood withdrawal. Consequently, serum of in total 8 NC, 18 PC, and 15 FC calves on day 14; 9 NC, 17 PC, and 20 FC calves on day 49 was analyzed. Here, a linear mixed model for repeated measures was performed (PROC GLIMMIX). The systematic part of the model (referred to as model 2) consisted of the following fixed effects:


(2) $$\begin{array}{l} \mu +{\text{Treatment}}_{\text{i}}+{\text{Batch}}_{\text{j}}+{\text{Parity}}_{\text{k}}+{\text{Sample moment}}_{\text{l}}\\ +\left({\text{Treatment}}_{\text{i}}\times {\text{Parity}}_{\text{k}}\right)+\left({\text{Treatment}}_{\text{i}}\times {\text{Sample moment}}_{\text{l}}\right) \end{array}$$

In the same notation as before (see model 1), and additionally Sample moment_l_ = intended calf age at the sample moment (l = 14 or 49 days) as the main effect and a two-way interaction between treatment and sample moment. Furthermore, the age difference in days between the calf's age at the intended sample moment and the actual sample moment was added as co-variate among the fixed effects. Random calf effects were introduced to handle repeated measurement. Further procedures were similar to model 1, so interactions that were not significant (*P* ≥ 0.05) were excluded from the model, the model comprised a random effect for the interaction between treatment and batch, approximate F-tests were used (41) and significance was declared at *P* < 0.05 for all fixed effects, and subsequent pairwise comparisons were made according to the Tukey method.

#### Hematology

A total 16 out of 144 hematology profiles at either day 1, 14, or 49 were missing, as those plasma samples could not be collected due to issues at blood withdrawal. Consequently, hematology profiles of in total 9 NC, 17 PC, and 17 FC calves on day 1; 7 NC, 18 PC, and 15 FC calves on day 14; and a total of 10 NC, 16 PC, and 19 FC calves on day 49 were analyzed. A generalized linear mixed model was used for the analysis of data expressed as continuous proportions (e.g., hematocrit) using a beta distribution with logit link function, whereas other quantitative data were analyzed with an ordinary linear mixed model similar to model 2. All further procedures were identical to model 2, except that now sample moment consisted of three levels (i.e., 1, 14, and 49 days); thus, a first-order autoregressive model (based on the actual distance between time points) was adopted to introduce correlation in the model between repeated measurements on the same animal.

#### Metabolite and Hormone Concentrations

Among 12 out of 96 blood samples of calves, those at 14 and 49 days of age were missing, as those samples could not be collected due to issues at blood withdrawal. Consequently, hormone concentrations of 7 NC, 18 PC, and 15 FC calves on day 14, and 10 NC, 14 PC, and 20 FC calves on day 49 were included in the analysis. A total 43 out of 144 hair samples were too dirty or had too few materials for cortisol extraction. This resulted in 10 NC, 14 PC, and 18 FC samples on day 0, 5 NC, 11 PC, and 11 FC samples on day 21, and 10 NC, 8 PC, and 14 FC samples on day 56. Continuous data on plasma cortisol, plasma IGF-1, plasma insulin, and serum cholesterol concentrations were analyzed with a linear mixed model identical to model 2 and its corresponding procedures. The statistical model for hair cortisol concentrations was also identical to model 2, except that here sample moment consisted of three levels (i.e., 0, 21, and 56 days) thus a first-order autoregressive model (based on the actual distance between time points) was adopted to introduce correlation in the model between repeated measurements on the same animal.

#### Cow Health and Performance

Due to low prevalences of cows with high SCC and antibiotic treatments for mastitis or endometritis, these parameters were analyzed using Fisher's exact method for pairwise comparisons. Data on the metabolic status of cows were analyzed with a linear mixed model similar to model 1, although now the moment of sampling (i.e., number of days prepartum or postpartum) was added as co-variate among the fixed effects. Due to hemolysis incidences, two cows (i.e., one PC, one FC) were excluded from the prepartum data set and one FC cow was excluded from the postpartum data set.

Milk yield, milk composition, and the number of days until the first insemination were analyzed with a linear mixed model identical to model 1 and its corresponding procedures.

#### Microbiota

A total 5 NC, 12 PC, and 13 FC calves on day 7; 4 NC, 10 PC, and 10 FC calves on day 28; 6 NC, 13 PC, and 16 FC calves on day 49; and 2 NC, 9 PC, and 12 FC calves on day 66 were analyzed. The data were analyzed using unconstrained and constrained ordination analysis (PCA/RDA) of Hellinger transformed microbiota compositions. With the RDA we evaluated the statistical significance of factors (i.e., treatment, parity, batch, sample moment) affecting microbiota composition. The statistical significance of separate taxa, with respect to treatment and health factors, were then evaluated using beta-binomial regressions. In this analysis, the false discovery rate and the corresponding adjusted *P-*values were calculated using the Benjamini-Hochberg procedure. Significance was declared at adjusted-*P* < 0.1.

## Results

### Growth Rates of Calves

Calves had a mean birth weight (± SE) of 40.2 ± 0.8 kg (39.4 ± 1.7 kg, 41.1 ± 1.1. kg, and 39.9 ± 1.3 kg for NC, PC, and FC calves, respectively). The FC calves had a greater ADG in body weight compared to PC and NC calves during the first 7 weeks of life (*P* < 0.001). In terms of skeletal growth rates, FC calves had a greater increase in back length compared to PC calves (*P* = 0.01), but not compared to NC calves (*P* = 0.79). Moreover, FC calves tended to have a greater increase in heart girth compared to NC calves (*P* = 0.08), but not compared to PC calves (*P* = 0.17). No significant treatment differences were found for the increase in hip height (Table 3).

**Table 3:** Growth performance of three groups of calves with different types of cow-calf contact (calf age: 1–7 weeks).

	**No contact**			**Partial contact**			**Full contact**			***F-value***	***P-value***
	**Mean**	**SE**	**95% CI**	**Mean**	**SE**	**95% CI**	**Mean**	**SE**	**95% CI**		
ADG in body weight	0.72^a^	0.05	0.34, 0.87	0.75^a^	0.03	0.47, 1.02	1.03^b^	0.05	0.63, 1.35	20.02	<0.001
Increase in heart girth^1^	0.35	0.02	0.26, 0.41	0.38	0.05	0.26, 0.57	0.43	0.02	0.29, 0.62	3.14	0.06
Increase in back length^1^	0.25^ab^	0.04	0.10, 0.45	0.20^a^	0.03	0.05, 0.43	0.29^b^	0.02	0.14, 0.45	4.87	0.01
Increase in hip height^1^	0.25	0.02	0.19, 0.36	0.24	0.01	0.17, 0.36	0.27	0.01	0.14, 0.40	1.08	0.40

*ADG, Average daily gain in kg/day*.

1 *Skeletal growth parameters are expressed in cm/day*.

*Different superscripts within a row indicate a significant difference (P < 0.05) between treatments*.

At 6 months of age, no treatment differences in mean absolute body weight (± SE) were present, as FC calves weighed on average 198.6 ± 8.7 kg, PC calves weighed 192.6 ± 7.7 kg, and NC calves weighted 211.4 ± 6.9 kg (*P* = 0.33).

### Health of Calves and Antibiotic Use

The FC calves had an increased mean THS (± SE) of 4.3 (± 0.6) compared to NC calves (2.1 ± 0.4) (*P* = 0.02) but did not differ in mean THS from PC calves (3.2 ± 0.4) (*P* = 0.43). No differences were found between NC and PC calves (*P* = 0.18). The THS values varied from 0 to 11 for FC calves, 0 to 8 for PC calves, and 0 to 4 for NC calves. The prevalence of calves classified with clinical symptoms for each health variable included in the THS can be found in Table 4.

**Table 4:** Prevalence of calves (%) in three treatment groups with different types of cow-calf contact that were classified at least once with clinical symptoms for various health variables.

	**No contact**	**Partial contact**	**Full contact**
Ocular discharge	0.0^a^	16.7^a^	70.0^b^
Nasal discharge	50.0	55.6	85.0
Cough	40.0	44.4	40.0
Navel inflammation	10.0	27.8	30.0
Neonatal diarrhea	80.0	83.3	80.0
Fever	20.0	5.6	20.0

*For each variable, values represent the percentage of calves within a treatment group scored at least once with a score ≥ 2 for ocular discharge, nasal discharge, cough, or navel inflammation, score > 2 for fever (calf age 1–7 weeks) and score ≥ 2 neonatal diarrhea (calf age 1–4 weeks) on a 0–3 point scale [adapted from (28)]. Different superscripts within a row indicate a significant difference (P < 0.05) between treatments*.

In the first 7 weeks of life, 21% of all calves were treated with antibiotics (i.e., for pneumonia and navel inflammation). The prevalence of calves treated with antibiotics tended to be higher in FC calves compared to NC calves (6 out of 20 calves versus 0 out of 10 calves, *P* = 0.07). No differences in the prevalence of calves treated with antibiotics were found between FC and PC calves (4 out of 18 calves in the latter group, *P* = 0.72), or between NC and PC calves (*P* = 0.27). No neonatal or postnatal mortality occurred among the experimental calves in the first 7 weeks of life.

### Immunoglobulin Concentrations in Calves

Colostrum quality, as reflected by an overall mean colostral brix score (± SE) of 25.81 ± 0.74%, did not differ among treatments (*P* = 0.81). Similarly, mean (± SE) bovine IgG concentration in plasma after colostrum intake did not differ among treatments (FC: 24.59 ± 3.13 mg/ml; PC: 22.22 ± 2.32 mg/ml; NC: 24.63 ± 2.53 mg/ml) (*P* = 0.97). However, the seven FC calves that suckled their first colostrum before they were bottle-fed had greater mean bovine IgG levels (32.61 ± 6.2 mg/ml) than FC calves that received their first colostrum by bottle (18.38 ± 2.99 mg/ml) (*P* = 0.03). Prevalence of failure of passive transfer (defined as IgG < 10 mg/ml (42)) was 12.5% and occurred in 2 PC and 4 FC calves (of which 1 suckled the first colostrum meal), no treatment effect was found (*P* > 0.28). With respect to the THS, those six calves were not identified as outliers.

Furthermore, mean N-IgA, N-IgG, and N-IgM did not significantly differ among treatments, although mean N-IgG declined (*P* < 0.001) and N-IgM increased (*P* = 0.002) from day 14 to day 49 for all treatments (Table 5).

**Table 5:** Effect of type of cow-calf contact on natural autoantibodies (N-IgA, N-IgG, and N-IgM titers) titers, and metabolite and hormone concentrations measured in the blood of calves at two different ages.

**Variable**	**No contact**				**Partial contact**				**Full contact**				***F-value* ^a^**	***P-value* ^a^**
	**Day 14**		**Day 49**		**Day 14**		**Day 49**		**Day 14**		**Day 49**			
	**Mean**	**SE**	**Mean**	**SE**	**Mean**	**SE**	**Mean**	**SE**	**Mean**	**SE**	**Mean**	**SE**		
N-IgG, titer	8.23	0.49	6.90	0.28	7.97	0.22	6.77	0.28	8.23	0.36	6.28	0.30	0.76	0.48
N-IgA, titer	1.51	0.31	2.29	0.60	1.53	0.21	2.05	0.36	2.07	0.38	2.02	0.22	1.83	0.17
N-IgM, titer	2.53	0.38	2.88	0.45	2.92	0.30	3.91	0.47	2.66	0.34	4.31	0.27	2.76	0.10
Cholesterol, mmol/L	1.70	0.22	2.65	0.20	1.75	0.15	2.89	0.18	1.54	0.19	2.77	0.14	0.06	0.94
Cortisol, ng/mL	1.58	0.21	2.39	0.41	2.64	0.36	1.28	0.27	3.32	0.51	3.28	0.56	4.27	0.06
Insulin, μU/mL	23.20	10.09	22.81	9.29	28.56	4.61	24.06	6.25	19.77	3.48	30.63	5.91	0.71	0.53

*Ig (G, A, M): Immunoglobulin*.

a *F-value and P-value for treatment effect*.

### Hematology of Calves

**Figure 2:**
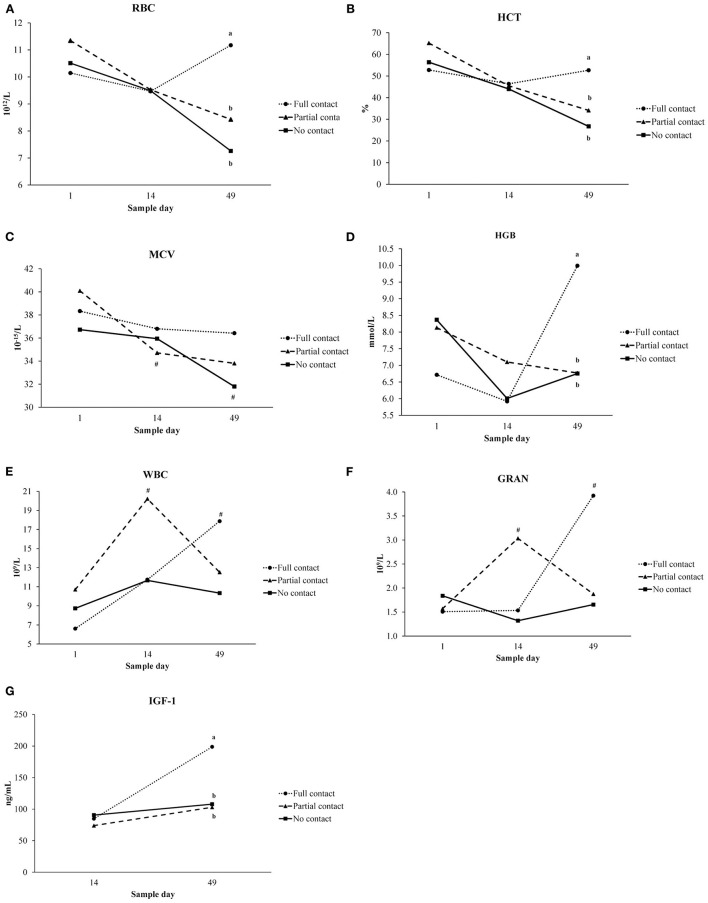
Interaction between treatment (no contact vs. partial contact vs. full contact) and sample moment (day 1, 14, 49) for hematological parameters (LSmeans) measured in plasma of dairy calves: **(A)** erythrocytes (RBC), **(B)** hematocrit (HCT), **(C)** mean corpuscular volume (MCV), **(D)** hemoglobin (HGB), **(E)** leukocytes (WBC), **(F)** granulocytes (GRA), and between treatment and sample moment (day 14, 49) for **(G)** insulin-like growth factor 1 (IGF-1). Different letters indicate significant differences (*P* < 0.05) between treatments within a sampling moment, # represent significant differences between sample days within a treatment group.

Interaction between treatment and sample moment was found for RBC, HCT, MCV, HGB, WBC, GRA values (Figure 2). On day 49, RBC, HCT, and HGB values were higher in FC calves than in NC calves (*P* ≤ 0.001). Only for NC and PC calves, MCV values significantly decreased from day 1 to day 49 (*P* < 0.001). WBC values were higher on day 49 than day 1 for FC calves, and higher on day 14 compared to day 1 for PC calves (*P* = 0.03). For FC calves, GRA values were higher on day 49 than day 1 (*P* = 0.01) (Figure 2).

No treatment effect was found for MCH, MCHC, PCT, LYM, MID, and PLT values (Table 6). For all treatments, MCH and MCHC were higher on day 49 than on day 1 and day 14 (*P* < 0.001), whereas PCT, LYM, MID, and PLT values were higher on day 14 and day 49 compared to day 1 (*P* ≤ 0.001). Similarly, no treatment effect was found for RDW and MPV values, but across treatments, those values were lower on day 14 and day 49 compared to day 1 (*P* < 0.001) (Table 6). The mean percentage of monocytes was higher in NC calves compared to PC and FC calves (*P* = 0.01) and was higher on day 49 compared to day 1 for all treatments (*P* = 0.01) (Table 6). Mean percentages of lymphocytes and neutrophils did not differ among treatments, although an effect of sample moment was found (*P* < 0.001). For all treatments, the percentage of lymphocytes was higher on day 14 and day 49 compared to day 1, and the percentage of neutrophils was lower on day 14 and day 49 compared to day 1 (Table 6).

**Table 6:** Effect of type of cow-calf contact on hematological parameters measured in plasma of calves at three different ages.

**Variable**	**No contact**						**Partial contact**						**Full contact**						***P-value^a^***
	**Day 1**		**Day 14**		**Day 49**		**Day 1**		**Day 14**		**Day 49**		**Day 1**		**Day 14**		**Day 49**		
	**Mean**	**SE**	**Mean**	**SE**	**Mean**	**SE**	**Mean**	**SE**	**Mean**	**SE**	**Mean**	**SE**	**Mean**	**SE**	**Mean**	**SE**	**Mean**	**SE**	
MCH, 10^−12^/L	0.78	0.21	0.60	0.04	1.13	0.35	0.63	0.01	0.62	0.02	0.74	0.03	0.60	0.01	0.64	0.02	0.99	0.19	0.85
MCHC, g/dl	39.13	10.16	32.35	2.44	68.85	22.43	30.43	0.59	32.69	1.04	41.84	1.87	30.41	0.58	33.76	1.61	53.15	10.83	0.63
RDW	5.77	0.63	3.97	0.30	3.65	0.60	6.19	0.27	4.93	0.39	4.09	0.48	5.82	0.45	4.27	0.17	4.35	0.18	0.52
PLT, 10^9^/L	464.20	78.68	603.44	79.91	479.38	39.12	361.00	24.46	650.17	69.41	683.40	151.36	330.91	32.83	662.46	65.89	546.87	43.00	0.34
MPV, 10^−15^/L	5.99	0.60	4.97	0.15	4.95	0.42	6.35	0.25	5.62	0.35	5.31	0.48	6.25	0.39	5.22	0.12	5.42	0.10	0.77
PCT, %	0.20	0.03	0.27	0.04	0.16	0.01	0.16	0.01	0.24	0.03	0.22	0.03	0.14	0.01	0.30	0.05	0.21	0.02	0.60
LYM, 10^9^/L	2.98	0.64	3.81	0.94	3.29	0.99	3.85	0.55	5.57	0.67	4.67	0.74	2.60	0.49	3.84	0.70	4.77	0.59	0.51
MID, 10^9^/L	4.79	1.19	6.92	2.22	5.40	1.92	6.56	1.60	10.05	1.41	10.58	2.38	3.40	0.65	8.30	1.97	8.66	1.70	0.54
BASO^*^, %	0.00	0.00	0.00	0.00	0.00	0.00	0.00	0.00	0.00	0.00	0.00	0.00	0.00	0.00	0.00	0.00	0.00	0.00	
EO^*^, %	0.67	0.37	1.00	1.00	0.50	0.34	0.29	0.17	0.17	0.12	1.25	0.51	0.56	0.29	0.13	0.13	0.84	0.32	
LYM, %	42.78	6.53	51.25	7.25	51.60	5.04	43.41	3.76	58.89	3.61	57.25	2.77	32.38	4.69	47.87	3.84	54.53	2.31	0.49
MONO, %	3.78	1.02	7.00	1.20	7.80	1.44	3.94	0.75	5.06	0.57	5.88	0.59	4.19	0.69	4.60	0.71	6.16	0.46	0.01
NEUT, %	50.56	6.70	40.88	7.87	40.10	4.83	52.35	5.18	35.89	3.59	38.38	3.17	62.88	4.68	47.40	3.83	38.47	2.43	0.42

*MCH, mean corpuscular hemoglobin; MCHC, mean corpuscular hemoglobin concentration; RDW, red cell width distribution; PLT, platelet count; MPV, mean platelet volume; PCT, procalcitonin; LYM, lymphocytes, MID, less frequently occurring and rare white blood cells; BASO, basophils; EO, eosinophils; MONO, monocytes; NEUT, neutrophils*.

a *P-value for treatment effect*.

* *The statistical model for this variable did not converge*.

### Metabolite and Hormone Concentrations of Calves

A significant interaction between treatment and sample moment was found for plasma IGF-1 concentrations, as FC calves had a higher mean IGF-1 concentration on day 49 compared to PC and NC calves (*P* < 0.001) (Figure 2). Furthermore, FC calves tended to have a higher mean plasma cortisol concentration (3.3 ± 0.38 ng/ml) compared to PC calves (2.05 ± 0.26 ng/ml) (*P* = 0.05) but did not differ from NC calves (2.06 ± 0.27 ng/ml) (*P* = 0.25). Mean serum cholesterol concentrations did not differ among treatments (*P* = 0.94), although it significantly increased from day 14 to day 49 for all treatments (*P* < 0.001). Mean plasma insulin concentrations did not differ among treatments (*P* = 0.53) or sample moment (*P* = 0.85) (Table 5). In addition, the mean hair cortisol concentration (± SE) was 7.67 ± 0.73 ng/g and did not differ between treatments (*P* = 0.29) or sample moments (*P* = 0.18) (Table 7).

**Table 7:** Effect of type of cow-calf contact on hair cortisol concentrations in dairy calves at three different ages.

	**No contact**						**Partial contact**						**Full contact**						***F-value***	***P-value* ^a^**
	**Day 0**		**Day 21**		**Day 56**		**Day 0**		**Day 21**		**Day 56**		**Day 0**		**Day 21**		**Day 56**			
	**Mean**	**SE**	**Mean**	**SE**	**Mean**	**SE**	**Mean**	**SE**	**Mean**	**SE**	**Mean**	**SE**	**Mean**	**SE**	**Mean**	**SE**	**Mean**	**SE**		
Cortisol, ng/g	7.93	1.74	3.41	0.73	3.20	0.55	9.72	2.07	8.42	1.42	8.15	3.79	9.38	2.15	10.55	3.22	4.80	0.44	1.26	0.29

a *P-value for treatment effect*.

### Cow Health and Performance

In the first 7 weeks of lactation 2 out of 10 NC cows, 5 out of 18 PC cows, and 6 out of 20 FC cows had at least once a high SCC (mean SCC > 200,000 cells/ml (43)) (*P* > 0.68). In total three FC cows were treated for mastitis with antibiotic injectors (*P* > 0.23). Furthermore, one NC and one FC cow were treated with non-steroidal anti-inflammatory drugs for endometritis (*P* > 0.36).

The metabolic status of the experimental cows during the dry period did not differ significantly among treatments, as reflected by an overall mean NEFA (± SE) (0.25 ± 0.03 mmol/L), BHBA (0.48 ± 0.02 mmol/L), urea (4.90 ± 0.27 mmol/L), magnesium (0.83 ± 0.02 mmol/L) and haptoglobin (0.17 ± 0.06 g/L) concentration (Table 8). In the postpartum period, mean BHBA (0.53 ± 0.03 mmol/L), urea (3.95 ± 0.26 mmol/L), and calcium (2.31 ± 0.02 mmol/L) did also not differ significantly among treatments (Table 8).

**Table 8:** Metabolic status of dairy cows during the dry cow period (2–21 days prepartum) and the fresh cow period (30–50 days postpartum) in three different cow-calf contact groups.

	**No contact**		**Partial contact**		**Full contact**		***F-value***	***P-value***
	**Mean**	**SE**	**Mean**	**SE**	**Mean**	**SE**		
**Dry cow period**								
NEFA, mmol/L	0.26	0.06	0.22	0.05	0.27	0.06	0.04	0.96
BHBA, mmol/L	0.50	0.04	0.46	0.05	0.47	0.04	0.25	0.79
Urea, mmol/L	4.69	0.39	5.23	0.53	4.70	0.40	0.56	0.58
Magnesium, mmol/L	0.83	0.03	0.84	0.04	0.82	0.04	0.57	0.58
Haptoglobin, g/L	0.10	0.02	0.21	0.12	0.17	0.09	0.07	0.93
**Fresh cow period**								
BHBA, mmol/L	0.54	0.04	0.54	0.07	0.53	0.03	0.73	0.49
Urea, mmol/L	3.36	0.61	4.79	0.52	3.65	0.31	3.29	0.05
Calcium, mmol/L	2.31	0.05	2.34	0.04	2.30	0.02	0.64	0.53

*NEFA, Non-esterified fatty acids; BHBA, Beta-hydroxybutyrate*.

The FC cows produced less milk in the milking parlor (mean daily yield ± SE: 17.01 ± 1.97 kg/d) throughout the first 7 weeks postpartum compared to PC (28.94 ± 1.1 kg/d) and NC cows (29.25 ± 2.25 kg/d) (*P* < 0.001). Moreover, milk of FC cows had a lower mean fat content (3.51 ± 0.13%) in contrast to PC (4.29 ± 0.14%) and NC cows (4.34 ± 0.15%) (*P* < 0.001). Similarly, FC cows tended to have a lower mean lactose content (4.26 ± 0.08%) compared to PC (4.50 ± 0.03%) and NC cows (4.55 ± 0.05%) (*P* = 0.07), although mean protein content did not differ among treatments (FC: 3.52 ± 0.09%, PC: 3.38 ± 0.05%, NC: 3.42 ± 0.06%; *P* = 0.32). Last, the mean of days open until first insemination (± SE) did not differ among treatments (FC: 74 ± 5, PC: 74 ± 3, NC: 70 ± 5; *P* = 0.47).

### Microbiota Composition

**Figure 3:**
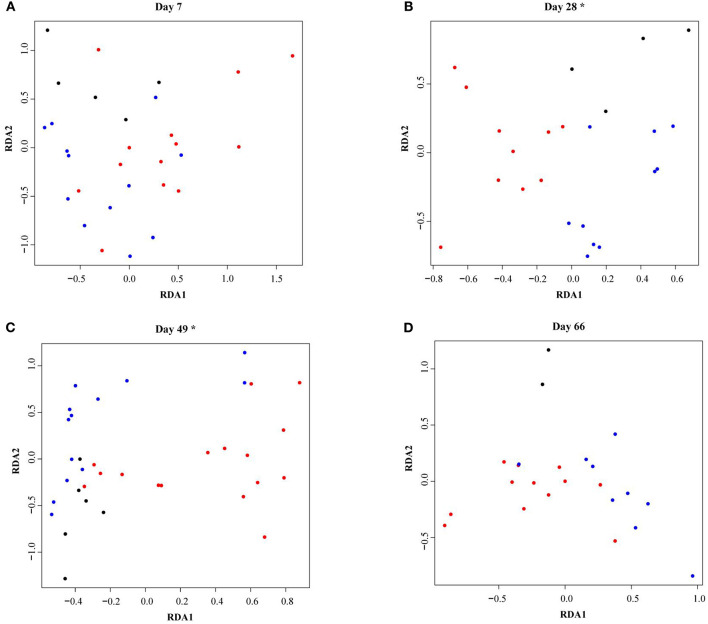
Redundancy analysis (RDA) of fecal microbiota in dairy calves reared with different types of cow-calf contact at **(A)** day 7, **(B)** day 28, **(C)** day 49, **(D)** day 66. The RDA is fitted conditioned on the batch effect. Individual calves with no contact are represented by black dots, partial contact by blue dots, and full contact by red dots. Asterisks indicate significant differences (*P* < 0.05) between treatment groups within a sample moment as evaluated with a permutation test.

As calves aged, fecal microbiota alpha-diversity increased (see Supplementary Figure 1) and the microbial community composition changed (see Supplementary Figure 2). In total 35% of the variance was explained by calf age and 28% by the individual calf. Fecal microbiota RDA analysis showed that calves reared with different types of CCC were distinctly grouped at 28 (*P* = 0.002) and 49 days of age (*P* = 0.01), but not at 7 (*P* = 0.45) or 66 (*P* = 0.18) days of age (Figure 3). On day 28, the fecal microbiota composition was different in FC calves compared to NC (*P* = 0.02) and PC calves (*P* = 0.001), although NC and PC calves did not differ (*P* = 0.19). Likewise, the fecal microbiota composition in FC calves differed from NC (*P* = 0.01) and PC calves (*P* = 0.02) on day 49, while NC and PC calves did not differ (*P* = 0.82).

Univariate analysis identified differences in relative abundances between the three CCC groups on the different sample moments based on false discovery rate-corrected *P*-values (Supplementary Table 1). On day 7, NC calves had a higher abundance of *Anaerotignum* and *CAG-81* compared to PC (adjusted-*P* = 0.09) and FC calves (adjusted-*P* = 0.02). Besides, FC calves had a greater abundance of *Lactobacillus B* compared to NC and PC calves (adjusted-*P* = 0.06) (see Supplementary Figure 3A). On day 28, FC calves had a higher abundance of *Lactobacillus B* compared to NC (adjusted-*P* = 0.09) and PC calves (adjusted-*P* =0.04) but had a reduced abundance of *Ruthenibacterium, Alistipes A, Barnesiella, Marseille-P3106, Parabacteroides, Odoribacter, Pseudoflavonifractor*, and *Clostridium-P* compared to NC (adjusted-*P* < 0.1) and PC calves (adjusted-*P* < 0.03) (see Supplementary Figure 3B). On day 49, FC calves had a higher abundance of *Butyricimonas* than NC (adjusted-*P* = 0.05) and PC calves (adjusted-*P* = 0.03) but had a reduced abundance of *Anaerotruncus, Ruminiclostridium C, Muribaculum, Ruminococcus E, RC9, Sphaerochaeta*, and *S5-A14-a* compared to NC (adjusted-*P* < 0.05) and PC calves (adjusted-*P* < 0.07) (see Supplementary Figure 3C). Given that on day 66 only 2 NC calf samples were included in the analysis, differences in relative abundances were identified between PC and FC calves only. The FC calves had a higher relative abundance of *Romboutsia, Turicibacter, Acholeplasma C, Acetivibrio*, and *Akkermansia* (adjusted-*P* < 0.09), and a reduced relative abundance of *CAG-873, Eubacterium F, Fournierella, Coprococcus B, Sphaerochaeta*, and *Barnesiella* compared to PC calves (adjusted-*P* < 0.09) (see Supplementary Figure 3D).

After correcting the microbiota data for treatment and sample moment, the effects of health and growth parameters on taxa were assessed (see Supplementary Table 2). Calves that suffered from respiratory issues had a higher abundance of *Olsenella* and *Slackia* compared to calves without respiratory issues (adjusted-*P* = 0.1). No differences in relative abundances were found for calves suffering from neonatal diarrhea (adjusted-*P* > 0.5) or navel inflammation (adjusted-*P* > 0.2) compared to calves without clinical symptoms for those diseases. In addition, no effect of weight gains (adjusted-*P* > 0.17) or absolute body weight (adjusted-*P* > 0.79) on relative abundances was found.

## Discussion

The objective of this study was to evaluate the effect of different types of CCC on the health status, blood parameters, fecal microbiota, and performance of dairy calves and cows. Our results showed that FC calves appeared to have more health issues, as reflected by an increased THS and a tendency for higher antibiotic usage in the first 7 weeks of life compared to NC calves. This was supported by elevated levels of RBC, HCT, and HGB on day 49 in FC calves compared to NC calves, and elevated WBC and GRA levels in FC calves on day 49 compared to day 14. Fecal microbiota composition changed as calves aged, and differences in relative abundances of various genera were found in FC calves compared to NC and PC calves. Furthermore, FC calves had a greater body weight gain than NC and PC calves in the first 7 weeks of life, which was accompanied by higher IGF-1 concentrations on day 49 in FC calves. In this study cow health was not affected by the type of CCC, although FC cows had, as expected, a lower daily machine-milked milk yield accompanied by a lower fat percentage in the first 7 weeks of lactation compared to NC and PC cows.

Ensuring adequate transfer of immunoglobulins is crucial to get off to a good start for the newborn calf (44). In our study, quick administration of the first colostrum meal by bottle assured comparable serum IgG concentrations for all calves. Interestingly, compared to bottle-feeding, suckling colostrum from the dam has been found to increase the amount of IgG absorbed by the calves, suggesting that suckling in itself may promote passive transfer (45, 46). FC calves that accidentally suckled their first colostrum may have benefited from this effect (in addition to having been able to ingest a higher amount of colostrum). Furthermore, bacterial contamination of colostrum during harvesting and feeding can interfere with immunoglobulin absorption, as bacteria originating from harvesting or storing colostrum may bind free immunoglobulins in the gut lumen or directly block uptake and transport of immunoglobulin molecules across intestinal epithelial cells (42). Research showed that harvesting colostrum into a bucket resulted in substantially higher bacterial counts than in directly stripped colostrum (which a suckling calf would be expected to obtain) (47). However, calves left to suckle their dams often risk failure of passive transfer, possibly due to delayed intake or intake of inadequate volumes of colostrum (48, 49). Therefore, close monitoring of colostrum intake in all systems including full CCC systems is highly recommended. As calves aged, immunoglobulin titers did not differ among treatment groups, although we found an increase of N-IgM titers and a decline of IgG titers from day 14 to day 49. Similar patterns were found in previous research (50). From 42 days of age onwards, calves are expected to have developed their own adaptive immunity (51). Our findings, therefore, suggest that all calves were able to exhibit sufficient endogenous production of immunoglobulins over time, regardless of CCC treatment. Hematological parameters are also known to change as calves age (52). Hence, the decrease in MCV and neutrophils accompanied by an increase in MCH, MCHC, PLT, lymphocytes, and monocytes over time imply a normal physiological development (52, 53). Correspondingly, the overall increase in cholesterol concentrations from day 14 to day 49 reflects the maturation of the gastrointestinal tract (54).

Besides the risk for failure of passive transfer, housing conditions are a major hazard for the health of the newborn calf (55). In the present study, three common calf disorders, namely umbilical cord infections, diarrhea, and respiratory issues (56), explain the increased THS in FC calves. Maternity pens are the first place where calves can be infected with pathogens (57). A recent review reported inconsistencies among studies that compared no contact vs. full contact with the dam in the first few days postpartum, because either beneficial, detrimental, or no effects of full contact on calf health were found (23). In our study, the relatively high prevalence of umbilical cord infection in PC and FC calves may have been the result of the postnatal housing conditions, as those cow-calf pairs remained in the maternity pen for the first 3 days postpartum to strengthen the cow-calf bond, whereas NC calves were moved to an individual calf box away from the dam directly after birth. Possibly, the prolonged residence time in the maternity pen posed a challenge on the management in terms of pen hygiene, which in itself is known to increase the risk for umbilical cord infection (56). Those infections are harmful to the general condition and health of the calf, as bacteria can migrate to joints, lungs, and other organs, and therefore pose a risk for enteric and pulmonary infections later in life (58). Thus, adequate maternity pen management and overall cleanliness of the calving area are of critical importance for CCC systems (23).

Diarrhea is mainly caused by inadequate management related to hygiene, housing, and feeding (59, 60). Previous work on prolonged CCC with suckling pointed to beneficial or no effects on calf diarrhea (23), although one study also found more health problems in FC calves mainly resulting from higher diarrhea incidences (61). Our study showed a high prevalence of neonatal diarrhea in all treatment groups, but we could not differentiate between infectious diarrhea or nutritional diarrhea. Perhaps, NC and PC calves were exposed to pathogens arising from milk feeding management (59), whereas diarrhea incidences in the FC calves may have been caused by the large amounts of milk that they consumed (61). Nevertheless, FC calves might also have been exposed to enteric pathogens due to the group housing and contact with floors that were contaminated with adult cow manure in the FC pen (62).

The risk for respiratory disorders in young calves increases when exposed to inadequate barn climate in terms of, for example, temperature, humidity, wind speed (draft) and air quality, and inappropriate (in particular wet) bedding (63). The high prevalence of ocular and nasal discharge accompanied by the tendency for increased use of antibiotics in FC calves compared to NC calves in the first 7 weeks of life likely reflected an increased incidence of respiratory disorders. This corresponds with the increased RBC, HCT, HBG, WBC, GRA values found in FC calves on day 49, an age when respiratory disorders in calves are common (40). Respiratory disorders can trigger an increase in erythropoietin production, which increases the number of erythrocytes and results in elevated RBC, HCT, HBG values (53). Moreover, leukocytes play an essential role in immune defense, and increasing levels of WBC and GRA can be indicative of inflammation (53). The FC calves were housed together with the cows and with calves of different ages, in groups of varying sizes, and in a pen that had open sidewalls. In contrast, NC and PC calves were pair housed in their own calf box that reduced contact with other animals, and that may have protected them from unfavorable climatic conditions, like draft (64). Given that existing cow pens are originally designed for adult animals rather than young calves, the potentially non-optimal climate in those pens can pose a challenge for calf health (19). Other studies on prolonged full CCC showed beneficial or no negative effects on calf health (61, 65–67). These inconsistencies might be due to the variability in study type and methodology, as the majority of those studies did not investigate calf health as a primary outcome measure, had different barn designs, and sometimes had small sample sizes. Moreover, the present experiment was conducted on a dairy farm with no previous experience with prolonged CCC. Successfully adopting new farm management systems depends, among others, on inner motivation, former experience with change, and the period of time over which new practices are implemented (68). Farmers that transformed their calf rearing system from a conventional to a full CCC system reported calf health benefits (17, 67, 69), but also acknowledged that it required additional infrastructure (69) and that it took time to reach the necessary change of perception on calf monitoring (17). We highly recommend future research focus on identifying and optimizing suitable cow-calf housing systems and managerial changes to ensure optimal calf health in CCC systems both during and after the transition period.

Despite the higher disease incidences in FC calves, we found a greater ADG in FC calves compared to NC and PC calves throughout the milk feeding period. However, FC calves no longer differed in absolute body weight from PC and NC calves at 6 months of age. This is in contrast with other studies in which growth benefits during the suckling period were maintained for months after separation compared with separated calves (22). Although, recent work did also not detect differences in body weight among heifers reared with and without prolonged CCC when they entered their first lactation (70). Possibly, FC calves experienced a growth dip after weaning and separation (see text footnote^1^), as weaning calves at a relatively young age from high volumes of milk while being not yet fully adapted to the solid feed is a well-known challenge in full CCC systems (71, 72). High growth rates during the milk feeding period are commonly reported in calves that suckle their dam freely for a prolonged period of time (61, 67, 73). We aimed to feed NC and PC calves at a rather high feeding schedule, while still following the Dutch standard practice by applying gradual weaning up to 8 weeks of age. Nevertheless, the free suckling of the dam provided calves the opportunity to meet their natural requirement is estimated at a milk consumption at about 20% of their body weight (74, 75). This could explain the relatively high ADG despite the impaired health status. Besides, the higher plasma IGF-1 concentrations in FC calves, compared to PC and NC calves on day 49, reflect a greater body condition related to the higher energy intake and thus ADG (76, 77). Plasma IGF-1 concentrations are considered important for the development of the gastrointestinal tract (GIT), as in young goats increased IGF-1 concentrations due to high intake of protein and energy were accompanied by an increased rumen papillae size (77). Possibly, compared to calves that were fed with limited amounts of milk, calves reared in full CCC have a differently developed GIT attributable to *ad libitum* milk intake. Correspondingly, we found preliminary evidence that the succession of microorganisms colonizing the GIT was affected by the type of CCC, as FC calves had different relative abundances of fecal microbiota compared to NC and PC calves. Similar results were found in 4-week old dairy calves reared with maternal contact compared to conventionally reared calves (78). That study reported higher relative abundances of Lactobacillus at day 28 in calves reared with maternal contact, which is in line with our findings in FC calves that had a higher relative abundance of *Lactobacillus* on both day 7 and day 28 compared to NC and PC calves. Early colonization of *Lactobacillus* spp. can provide probiotic effects for calves and offer protection against neonatal diarrhea (79). However, given that conventionally reared calves in (78) were fed with waste-milk that contained residuals of antimicrobials, the changes in microbiota composition could not be attributed solely to maternal contact in that study. Nonetheless, our exploratory results imply that microbial communities developed distinctively between FC and PC calves at 28 and 49 days of age, perhaps because full CCC allowed for more vertical transmission of microbes via the dam (i.e., unimpeded reciprocal licking, contact to adult feces, and exposure to microbes on the teat skin and in maternal milk) (80, 81). Changes in microbial communities can also be affected by age, diet, antibiotics, and environmental factors (24, 82). The FC calves were suckling *ad libitum* milk from their dams and were housed inside the cow pen, whereas NC calves were housed in a separate calf barn, PC calves were housed adjacent to the cow pen, and those two groups were fed tank milk via teat buckets. Yet, we found that alpha-diversity increased in calf fecal microbiota as animals aged, but no treatment effect on fecal microbiota composition was found in the first week (i.e., day 7). This corresponds to previous work that reported increasing diversity in GIT microbial communities as pre-weaned calves aged, except on day 7 indicated by similar microbiota in various GIT regions (83). Given that we found no treatment effect after weaning (i.e., day 66) as well, implies that pre-weaning diet was an important factor explaining the differences in colonization (84, 85). Antibiotic treatments are known to affect the fecal microbiota composition (86), nevertheless, we did still detect differences in fecal microbial communities between PC and FC calves even though they did not differ in the number of antibiotic treatments. Future studies are needed to enhance our understanding of maternal factors (as well as external factors) that may affect dairy calf microbiota, and of the clinical and biological relevance of these effects.

In line with the high body weight gains of FC calves, FC cows had a lower machine-gained milk yield compared to NC and PC cows. *Ad libitum* milk consumption by calves is known to reduce machine-gained milk yield (66, 87, 88). Besides, we found a reduced milk fat content in harvested milk of FC cows, which is likely to be caused by impaired alveolar milk ejection during the milking process due to suckling (19, 87, 89). Reduced amounts of saleable milk with decreased fat content during the suckling period could negatively impact farm income in full CCC systems (19, 90). However, any reduction in saleable milk income can only truly be considered a loss if the milk intake suckled by calves exceeds the costs of what they would have been fed through other methods (e.g., bucket feeding with milk replacer, bulk tank milk, or waste milk) (22). In addition, FC cows tended to have a decreased lactose content compared to NC and PC cows, which may be explained by the few cases of high SCC incidences. Lactose content tends to decrease when SCC increases due to clinical or subclinical udder inflammation (91). Yet, the type of CCC did not negatively affect cow health in the present study, which is in agreement with previous work that assessed udder health in suckled and non-suckled dams (67, 92, 93). Interestingly, our study showed that the metabolic status of the cows was not affected by suckling, even though twice-daily milking in addition to *ad libitum* suckling was expected to increase the metabolic stress in early-lactating cows (94). Previous work suggested that three times a day machine-milking of suckled cows resulted in a severe negative energy balance, as expressed by a heavyweight loss, elevated NEFA concentrations, and decreased glucose concentrations in their blood compared to non-suckled cows that were milked either three or six times a day (95). Blood metabolites in our study were similar to those in a recent study on cow serum metabolites (96), although our relatively low urea concentrations in early lactation may be a result of low protein content in the feed among other management factors (97). Despite the large sampling interval used in this study, individual variation in sampling times was equally distributed among the treatment groups (both pre- and postpartum) and was thus not expected to affect the results.

Given that the standard practice of early cow-calf separation is perceived as contentious by part of the public for ethical reasons (14, 15) and that rearing calves in full CCC with their dam does not necessarily guarantee an adequate calf health status, partial CCC might be considered as a feasible compromise for rearing dairy calves. Such a rearing system has the potential to increase the social acceptance of the dairy sector by allowing limited cow-calf interactions (98), while at the same time meeting producers' concerns regarding calf health and the amount of harvested milk (18). Because stockmanship and housing conditions are crucially related to calf health (4, 99), we strongly recommend efforts that identify the key features of best practices in full CCC systems to safeguard calf health and enable a successful transition for farmers interested in this calf rearing system. Moreover, since full CCC may provide longer-term benefits for calves' behavioral development (22), we believe that longitudinal studies into the effects of CCC beyond the time-frame of the current experiment are also warranted.

## Conclusion

This study shows that rearing calves in full contact with their dam compromised calf health in the first 7 weeks of life, as reflected by more health issues, elevated hematological parameters, and a tendency for higher antibiotic usage compared to calves reared without contact with the dam. In comparison with partial or no contact, full contact resulted in a greater average daily body weight gain and in a different calf fecal microbiota composition with, so far, unknown biological implications. Cow health was not affected by the type of cow-calf contact. Cows that were suckled by their calves had a lower machine-gained milk yield and a lower milk fat content compared to cows in a partial or no contact system.

## Data Availability Statement

The raw data supporting the conclusions of this article will be made available by the authors, without undue reservation.

## Ethics Statement

The animal study was reviewed and approved by AVD4010020174307.

## Author Contributions

MW, CV, EB, DO, AK, and CR were involved in the experimental design of the study. MW and CV executed the project and collected data. MW, EB, RB, JG, DB, GG, and CR analyzed the data. MW, CV, EB, RB, JG, AK, GG, DO, DB, and CR interpreted data. MW drafted the initial manuscript. CV, EB, RB, JG, GG, AK, DB, DO, and CR provided critical revision and improvements. All authors approved of the final version of this manuscript and agree to be held accountable for the content therein.

## Funding

This study was financed by DairyNL (ZuivelNL; organization of the Dutch dairy supply chain, The Hague, the Netherlands) and the Dutch Ministry of Agriculture, Nature and Food Quality (LNV, The Hague, the Netherlands) as part of the research program One Health for Food (1H4F, The Hague, the Netherlands). The contribution to this article by EB was supported by the ERA-net CORE Organic Cofund project GrazyDaiSy (ID 1871).

## Conflict of Interest

The authors declare that the research was conducted in the absence of any commercial or financial relationships that could be construed as a potential conflict of interest. The handling Editor declared a past co-authorship with one of the authors, EB.

## Publisher's Note

All claims expressed in this article are solely those of the authors and do not necessarily represent those of their affiliated organizations, or those of the publisher, the editors and the reviewers. Any product that may be evaluated in this article, or claim that may be made by its manufacturer, is not guaranteed or endorsed by the publisher.

## Footnotes

^1^Wenker ML, van Reenen CG, Bokkers EAM, McCrea K, de Oliveira D, Sorheim K, et al. Comparing gradual debonding strategies after prolonged cow-calf contact: stress responses, performance, and health of dairy cow and calf. *Appl Anim Behav Sci*. (under review).
