# Supplementary material for: Effect of Type of Cow-Calf Contact on Health, Blood Parameters, and Performance of Dairy Cows and Calves
Source: Front Vet Sci. 2022 Apr 12;9:855086. doi: 10.3389/fvets.2022.855086 (PMC9039747; doi:10.3389/fvets.2022.855086)
Supplement: Supplementary file 1 [file Data_Sheet_1.DOCX]

Supplementary Material S1

# Detailed methods on the bioinformatic analysis of calves’ fecal microbiota

## Library construction and sequencing

Sequence processing and preparational statistical analyses were performed in R 4.0.2 (1). The amplicon sequences were demultiplexed and subsequently filtered, trimmed, error-corrected, dereplicated, chimera-checked, and merged using the dada2 package (v.1.16.0, (2)). By using the standard parameters except for TruncLength=(265, 235), trimLeft=(35,35), maxEE=2, and minOverlap=10. Reads were classified using the naïve Bayesian classifier and the Genome Taxonomy Data Base (GTDB v86; (3)). Using R package phyloseq (4), the ASVs were aggregated to the Genus level. The data were filtered for low abundant taxa separately per day. Taxa that were present in at least half of the calves of a particular day were selected for that day. After filtering, the library size ranged from 43204-173453, 35888-148311, 4535-30133, and 35611-124957 for, respectively, day 7, 28, 49, and 66 (Supplementary Figure 1). For these days we selected 34, 81, 72, and 111 taxa containing 25%, 22%, 27%, and 21% zeroes, respectively.

## **Statistical analysis**

The data were explored using unconstrained and constrained ordination analysis (PCA/RDA) of Hellinger transformed microbiota compositions using R package vegan (5). Significance was assessed using the permutation test for the RDA.

We first fitted a PCA across all days (using a set of 86 taxa that are selected for at least two days), to conclude that the day effect is the primary source of variation. Further assessment of the effect of parity, batch and treatment was done separately per day, using only the taxa selected for that day. Parity had little effect (P-values > 0.05 for all days) and could be ignored. Batch also has little effect (P-values > 0.05 for all days), but for interpretational purpose was included in evaluation of the treatment effect and health variables.

The effect of treatment on the separate taxa was evaluated using beta-binomial regression. In the beta-binomial regression one model is fitted per taxon using treatment as explanatory variable; the batch effect was also included in the model. These models were fitted per day using R package corncob (6). To account for the small sample size (and the resulting possible type I error inflation), the parametric bootstrapped was used (1e4 bootstrap samples) to estimate the P-value. Using these P-values, the false discovery rates (FDR) were calculated using the Benjamini-Hochberg procedure.

The health variables were also evaluated using beta-binomial regression. Based on the weekly health assessments, calves diagnosed with clinical symptoms for navel inflammation (i.e. navel score ≥ 2), neonatal diarrhea (i.e. feces score ≥ 2), and respiratory issues (i.e. a composite respiratory score ≥ 4 (based on the sum of ocular discharge, nasal discharge, cough score)) (Table 2). The health parameters were evaluated across days; analysis per day was not possible due to the low occurrence of some health variables. In this analysis we used a more restrictive set of taxa, only including those that were selected in at least three days (48 taxa). The health variable were included as a covariate in a model that also included calf and batch as (nested) random effects, and treatment and sample day as fixed effect. These models were fitted using R package glmmTMB (7), which allows including random effects in the beta-binomial regression.

# Supplementary figures

# (A)

#
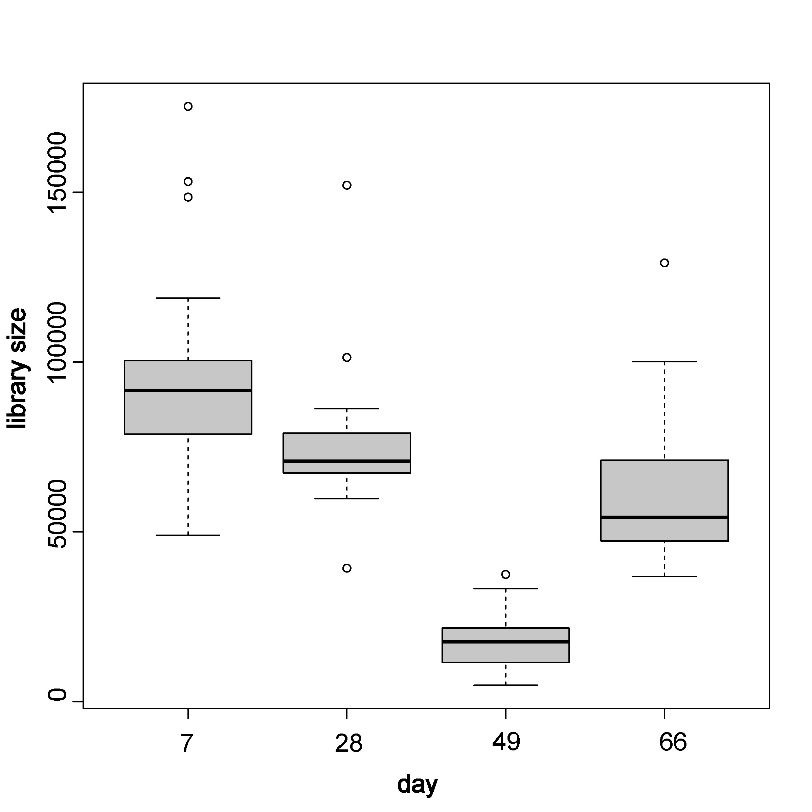


# (B)

#
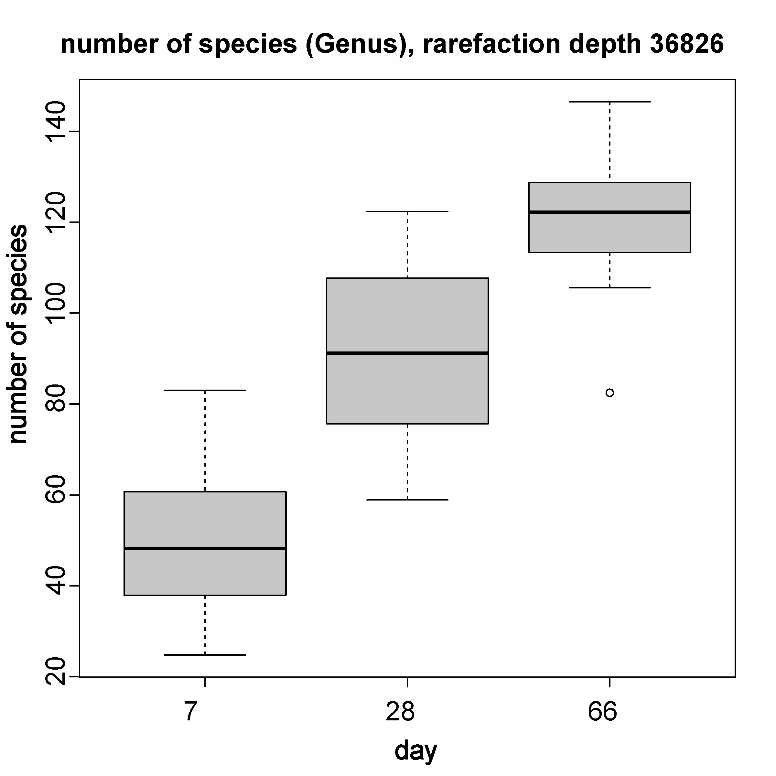


# Supplementary Figure 1. Boxplot diagrams for (A) library sizes and (B) microbiota alpha-diversity per sample moment.


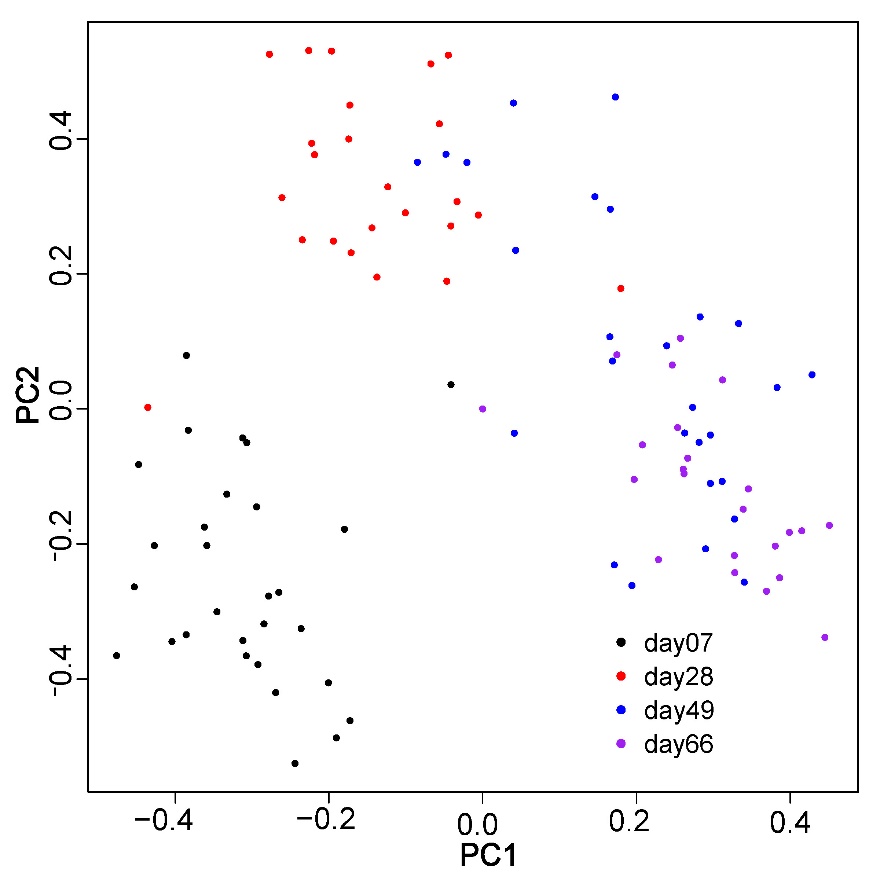


**Supplementary Figure 2.** PCA for all fecal samples (includes taxa selected on ≥ two days, Hellinger transformed, the PCA is conditioned on calf). Per day, each dot represents an individual calf. In total 35% of the variance is explained by day and 28% by the individual animal.

# (A)

#
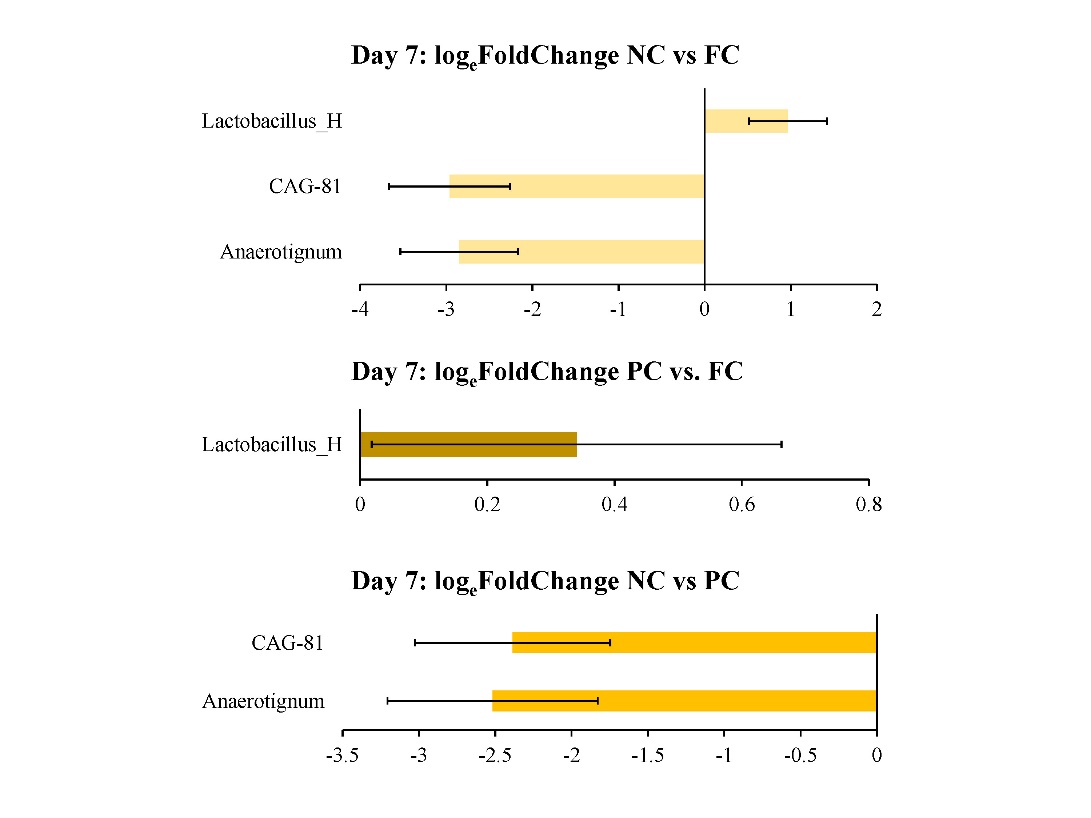


**(B)**


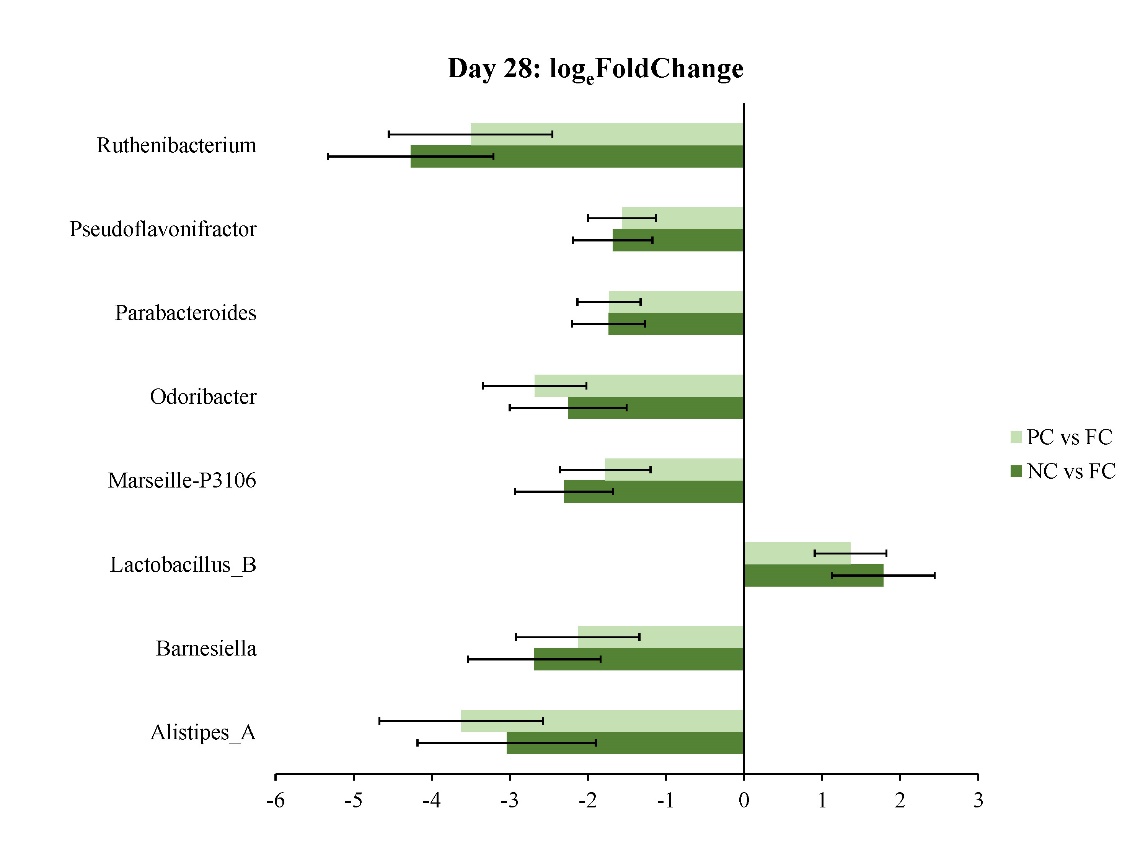


**(C)**


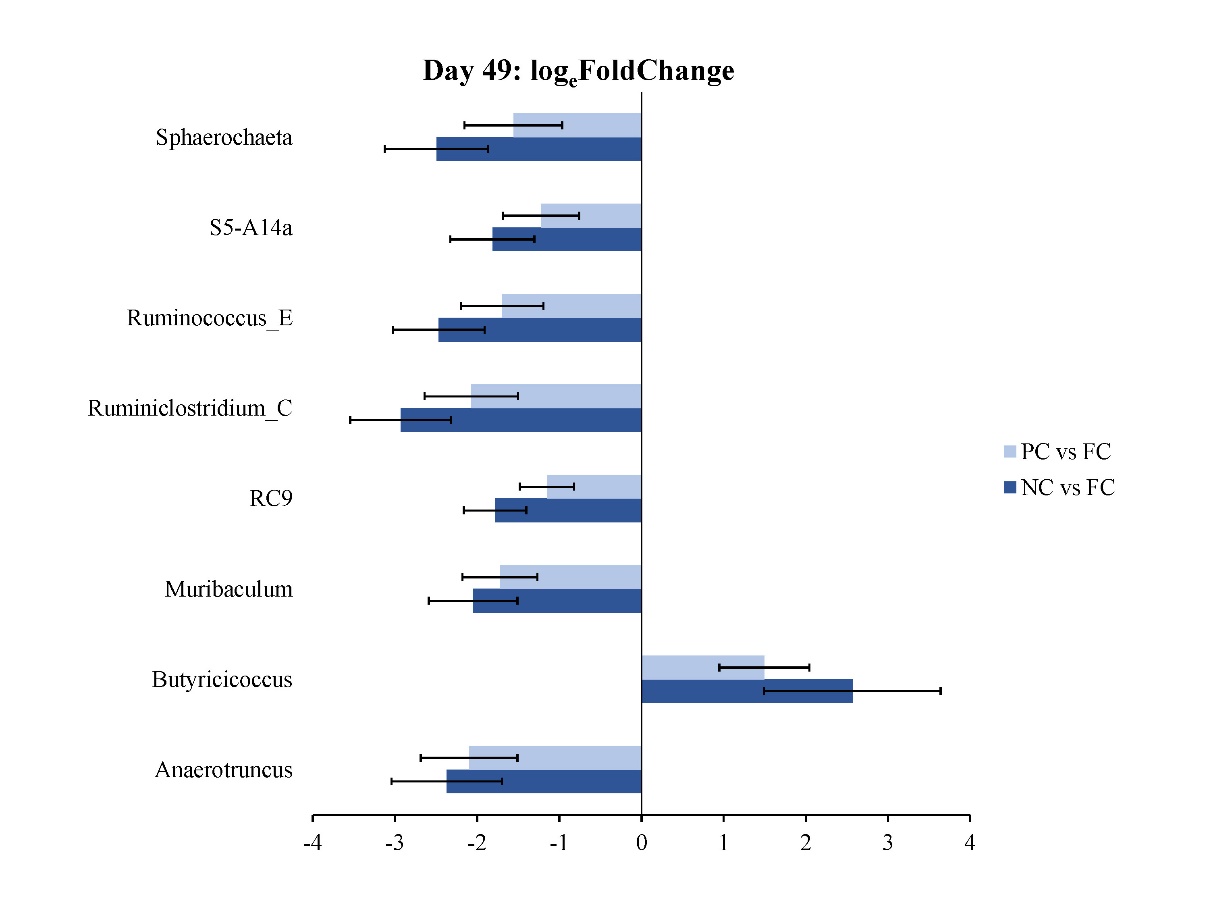


**(D)**


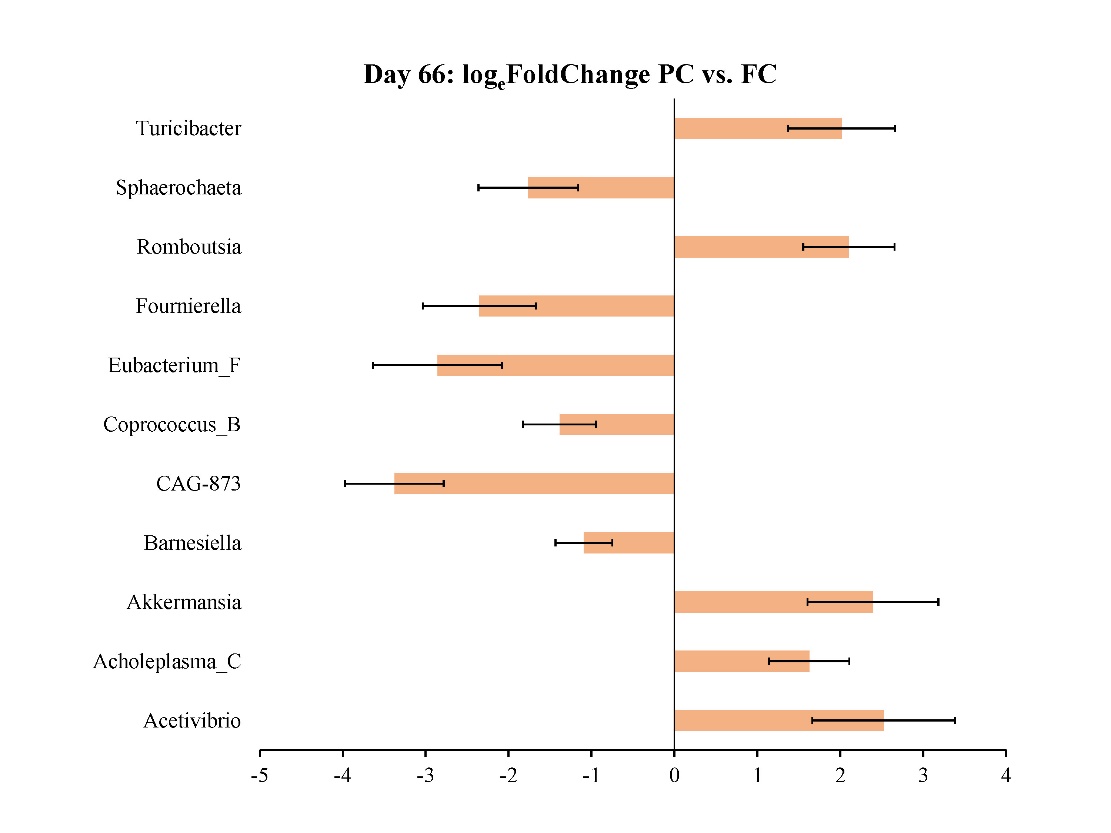


**Supplementary Figure 3.** Differential abundances of ASVs between the different cow-calf contact groups (NC = no contact, PC = partial contact, FC= full contact) at different sample moments, **(A)** day 7, **(B)** day 28, **(C)** day 49, and **(D)** day 66. A negative value of natural log fold change (± SE) meant significantly more abundance in the control group (i.e. first mentioned group) for taxon. All taxa with a FDR < 0.10 are displayed.

# Supplementary tables

**Supplementary Table 1.** Excel file with taxa in calf feces, including fold change estimates, 95 % CI, natural log fold change estimates, SE natural log fold change, P-values, and FDR-adjusted P-values of pairwise comparisons for treatments per day.

**Supplementary Table 2.** Excel file with taxa in calf feces, including fold change estimates, 95 % CI, p-value and FDR-adjusted P-values for various calf health and growth variables corrected for treatment and day.

# References

1. R Core Team. R: A language and environment for statistical computing. (2021) Available at: https://www.r-project.org/

2. Callahan BJ, McMurdie PJ, Rosen MJ, Han AW, Johnson AJA, Holmes SP. DADA2: High resolution sample inference from Illumina amplicon data. *Nat Methods* (2016) **13**:581. doi:10.1038/NMETH.3869

3. Parks DH, Chuvochina M, Rinke C, Mussig AJ, Chaumeil P-A, Hugenholtz P. GTDB: an ongoing census of bacterial and archaeal diversity through a phylogenetically consistent, rank normalized and complete genome-based taxonomy. *Nucleic Acids Res* (2021) doi:10.1093/NAR/GKAB776

4. McMurdie PJ, Holmes S. phyloseq: An R Package for Reproducible Interactive Analysis and Graphics of Microbiome Census Data. *PLoS One* (2013) **8**:e61217. doi:10.1371/JOURNAL.PONE.0061217

5. Oksanen J, Guillaime Blanchet F, Friendly M., Kindt R., Legendre P., McGlinn D., Minchin PR., O’Hara RB., Simpson GL., Solymos P., et al. The vegan package. Community Ecology Package. . (2007). Available at: https://r-forge.r-project.org/projects/vegan/ [Accessed November 19, 2021]

6. Martin BD, Witten D, Willis AD. Modeling microbial abundances and dysbiosis with beta-binomial regression. *Ann Appl Stat* (2020) **14**:94–115. doi:10.1214/19-AOAS1283

7. Brooks ME;, Kristensen K;, Van Benthem KJ;, Magnusson A;, Berg CW;, Nielsen A;, Skaug HJ;, Machler M;, Bolker BM, Brooks ME, et al. glmmTMB balances speed and flexibility among packages for zero-inflated generalized linear mixed modeling. *R J* (2017) **9**:378–400. doi:10.3929/ETHZ-B-000240890
